# Troxerutin Stimulates Osteoblast Differentiation of Mesenchymal Stem Cell and Facilitates Bone Fracture Healing

**DOI:** 10.3389/fphar.2021.723145

**Published:** 2021-08-09

**Authors:** Xiao Yang, Jiang Shao, Xiao-Min Wu, Fei-Fei Pan, Shao-An Yang, Xiao-Hua Pan, An-Min Jin

**Affiliations:** ^1^ Department of Spinal Surgery, Zhujiang Hospital, Southern Medical University, Guangzhou, China; ^2^ Department of Orthopedics, Affiliated Hospital of Shandong University of Traditional Chinese Medicine, Jinan, China; ^3^ Department of Orthopaedics, The Second School of Clinical Medicine, Southern Medical University, The Second Affiliated Hospital of Shenzhen University, The Clinical Medical College of Guangdong Medical University, People’s Hospital of Shenzhen Baoan District, Shenzhen, China; ^4^ School of Pharmaceutical Sciences, Southern Medical University, Guangzhou, China; ^5^ Department of Traumatic Orthopedics, Zhujiang Hospital, Southern Medical University, Guangzhou, China

**Keywords:** Troxerutin, osteogenic differentiation, mesenchymal stem cells, bone fracture healing, Wnt/β-catenin signaling

## Abstract

Troxerutin (TRX), a semi-synthetic derivative of the natural bioflavonoid rutin, is a bioactive flavonoid widely abundant in various fruits and vegetables. Known as vitamin P4, TRX has been demonstrated to have several activities including anti-inflammation, anti-oxidants, vasoprotection, and immune support in various studies. Although rutin, the precursor of troxerutin, was reported to have a protective role against bone loss, the function of TRX in skeletal system remains unknown. In the present study, we found that TRX promoted osteogenic differentiation of human mesenchymal stem cells (MSCs) in a concentration-dependent manner by stimulating the alkaline phosphatase (ALP) activity, calcium nodule formation and osteogenic marker genes expression *in vitro.* The further investigation demonstrated that TRX stimulated the expression of the critical transcription factor β-catenin and several downstream target genes of Wnt signaling, thus activated Wnt/β-catenin signaling. Using a femur fracture rats model, TRX was found to stimulate new bone formation and accelerate the fracture healing *in vivo*. Collectively, our data demonstrated that TRX could promote osteogenesis *in vitro* and facilitate the fracture healing *in vivo*, indicating that TRX may be a promising therapeutic candidate for bone fracture repair.

## Introduction

Fractures are the most common orthopedic problems often caused by accidental damages. About seven million people suffered from fractures in the United States each year and the average citizen in the developed country may expect to sustain at least one fracture during their lifetime (Praemer et al., 1999). Delayed healing and nonunion of fracture are common phenomena in clinical practices. A prospective cohort study involving 736 patients with an open long bone fracture showed that nonunion occurred in 17% of the patients, and delayed healing occurred in 8% (Westgeest et al., 2016). More importantly, delayed healing and nonunion are more common phenomena in the elderly patients for they have a lower capacity of mesenchymal progenitor cell division and differentiation (Kasper et al., 2009; Foulke et al., 2016). Therefore, how to improve the bone healing and regeneration potential is imperative to the patients. Mesenchymal stem cells (MSCs), as a progenitor cell of osteoblast, have been widely used in bone regeneration (Chamberlain et al., 2007). And systemic and local administration of allogeneic bone marrow-derived MSCs promoted fracture healing in rats (Ghasroldasht et al., 2018). Stimulating osteogenic differentiation of MSCs may be a potential therapeutic strategy for bone repair and regeneration.

Traditional Chinese herbs have been used for thousands of years, and many natural products have been reported to reduce bone loss (Freires et al., 2017). Troxerutin (TRX) is a bioactive flavonoid widely abundant in various fruits and vegetables (Lu et al., 2011). As a semi-synthetic derivative of the natural bioflavonoid rutin, TRX is also known as vitamin P4, which has been demonstrated to exert anti-oxidant, anti-inflammatory, and anti-cancer activities (Farajdokht et al., 2017; Shu et al., 2017; Yang et al., 2018; Zamanian et al., 2021). The precursor rutin was reported to have a protective role against bone loss (Xiao et al., 2019), but its usage is very limited due to the poor aqueous solubility and low bioavailability (Zhang et al., 2010). Considering that TRX has high aqueous solubility and validated chemical stability, it may have more beneficial to preventing osteoporosis and promoting bone regeneration.

In the present study, it was demonstrated that TRX promoted the osteogenic differentiation of human MSCs *in vivo* and improved new bone formation and accelerated the fracture healing *in vivo*. Wnt/β-catenin signal transduction is crucial for maintaining the homeostasis of bone mass. It is well established that this signaling play a significant role in the regulation of osteogenic differentiation and bone development (Duan and Bonewald, 2016). Our further investigation showed that the expression of the critical transcription factor β-catenin and several downstream target genes of Wnt signaling was significantly up-regulated by TRX, which led to the activation of Wnt/β-catenin signaling. Therefore, TRX could promote osteogenesis *in vitro* and facilitate the fracture healing *in vivo via* activating Wnt/β-catenin signaling, indicating that TRX may be a promising therapeutic candidate for bone fracture repair.

## Materials and Methods

### Cell Culture and Induction of Osteogenesis

Human bone marrow-derived mesenchymal stem cells (MSCs) were isolated from bone marrow according to the previous studies (Zhang et al., 2011; Feng et al., 2018). Briefly, bone marrow was aspirated from a healthy 38 year-old male donor with formal consent and approval by the local ethics committee. The isolated cells were cultured in a-minimum essential medium (α-MEM, Invitrogen, Carlsbad, CA, United States), supplemented with 10% fetal bovine serum (FBS, Gibco, United States) and 1% penicillin-streptomycin (Gibco) in a humidified atmosphere containing 5% CO2 at 37°C. The osteogenic differentiation of MSCs was induced by the classical inducers including 10 nM dexamethasone (Sigma-Aldrich, St. Louis, MO, United States), 50 μg/ml ascorbic acid (Sigma-Aldrich), and 10 mM glycerol 2-phosphate (Sigma-Aldrich) as described previously (Sun et al., 2015). The differentiation medium was replaced every 3 days. TRX was purchased from Aladdin (Shanghai, China) and dissolved in 0.1% DMSO for usage 0.1% DMSO was used as control.

### Cell Viability Assays

The human MSCs were seeded in 96-well plate at density 1.5 × 10^4^ per well for 24 h, then treated with different concentrations of TRX (from 0 to 200 µM). After incubated for 24, 48, and 72 h, the methylthiazolyl tetrazolium (MTT) assays were performed. The MTT solution (0.5 mg/ml) was added, and after 4 h, the deposition was dissolved with 100 µL DMSO. The absorbance was measured at the wavelength of 570 nm using Spectramax Gemini dual-scanning microplate reader (Molecular Devices, United States).

### Alkaline Phosphatase Activity and Alizarin Red S Staining Assays

The human MSCs were treated with TRX in the osteogenic induction medium (OIM) for 7 days, then the cells were collected, qualitative, and quantitative examination of ALP activity were carried out according to the protocol of BCIP/NBT Alkaline phosphatase Color Development Kit (Beyotime, Shanghai, China). For the alizarin red S staining, the TRX-treated MSCs were incubated in OIM for 14 days, and washed with PBS twice and fixed with ice-cold 75% ethanol for 15 min. The MSCs were then stained with 2% Alizarin Red S staining solution (pH 4.2, Leagene, Beijing, China) for 30 min. The stained calcified nodules were dissolved with 10% Hexadecylpyridinium chloride monohydrate (Sigma-Aldrich) at room temperature and then the absorbance was detected at the wavelength of 562 nm.

### RNA Extraction and Quantitative Polymerase Chain Reaction Examination

Total RNA was extracted by the Animals Total RNA Isolation Kit (FOREGENE, Chengdu, China) according to the manufacture’s instruction. After the concentration and purity of total RNA were measured by Nanodrop (Thermo Fisher Scientific), cDNA was reversely transcribed from RNA samples by PrimeScript™ RT Reagent Kit (TaKaRa, Japan). The PowerUp™ SYBR™ Green Master Mix (Thermo Fisher Scientific) was applied for the qPCR examination using the LightCycler 480 system (Roche, Basel, Switzerland). The relative fold changes of candidate genes were analyzed by using the 2^−ΔΔCt^ method. The house-keeping gene GAPDH was served as internal control. Primer sequences used in qPCR examination were displayed in Table 1.

**TABLE 1:** Primers for qRT-PCR examination.

	Forward	Reverse
Runx2	GAC​AAG​CAC​AAG​TAA​ATC​ATT​GAA​CTA​CAG	GTA​AGG​CTG​GTT​GGT​TAA​GAA​TCT​CTG
OPN	CTGAAACCCACAGCCACA	TGTGGAATTCACGGCTGA
OSX	GCC​AGA​AGC​TGT​GAA​ACC​TC	TGA​TGG​GGT​CAT​GGT​GTC​TA
Cmyc	TTC​GGG​TAG​TGG​AAA​ACC​AG	CAG​CAG​CTC​GAA​TTT​CTT​CC
CD44	TCA​GAG​GAG​TAG​GAG​AGA​GGA​AAC	GAA​AAG​TCA​AAG​TAA​CAA​TAA​CAG​TGG
Survivin	AGG​ACC​ACC​GCA​TCT​CTA​CAT	AAG​TCT​GGC​TCG​TTC​TCA​GTG
GAPDH	TCC​ATG​ACA​ACT​TTG​GTA​TCG	TGT​AGC​CAA​ATT​CGT​TGT​CA

### Luciferase Assays

The hMSCs were seeded into six well at the density of 2 × 10^4^ for 24 h. Cells grown to 70–80% confluence were transiently transfected with TOPFlash and PGMLR-TK by using the Lipofactamine™ 3,000 Reagent (Invitrogen). About 24 h later, the cell culture medium that contains TRX (100 μM, 200 μM) was added. After 2 days incubation with TRX, the cells were harvested and lysed for measuring the luciferase activity with Bright-GloTM luciferase Assay System (Promega) following the manufacturer’s instruction.

### Western Blotting

Total protein was extracted by RIPA Lysis and Extraction Buffer (Thermo Fisher Scientific) supplemented with the protease inhibitor cocktail (Roche). And the nuclear and cytoplasmic protein were isolated by the Nuclear and Cytoplasmic Protein Extraction Kit (KeyGEN, Nanjing, China) according to the manufacturer’s instructions. The protein was qualified by the BCA assay kit (Thermo Fisher Scientific). Then the soluble protein was separated by SDS-PAGE (10%) and transferred to PVDF membranes. The membranes were then blocked with 5% skimmed milk and probed with the following antibody: β-catenin (1:1,000; Cell Signaling Technology, United States) or GADPH (1:2,000; Cell Signaling Technology, United States). After incubation with the appropriate secondary antibody conjugated with HRP (1:1,000 dilution), the chemiluminescence (ECL, Hangzhou, China) was applied to visualize the bands.

### Rat Femoral Fracture Model

Twenty-four Sprague-Dawley rats (male, 12-week-old) were purchased from the Laboratory Animal Research Centre, Southern Medical University. Animal ethics was approved by the Institutional Animal Care and Use Committee (IACUC) of Southern Medical University (Guangzhou, China). The modified rat closed transverse femoral fracture model was applied in this study (Nyman et al., 2009; Liang et al., 2019). Briefly, the surgery was carried under general anesthesia (40 mg/kg ketamine and 4 mg/kg xylazine) and sterile condition. A 2-cm incision was made in the lateral aspect of right thigh, the osteotomy was made with an electric saw at the middle site of the femur, and then the femur was fixed with Kirschner’s needle penetrated into medullary cavity. Animals were randomly divided into four groups (n = 6 per group): surgery without any treatment (Blank); femur fracture with 0.1% DMSO treatment group (Control); femur fracture with a low dose injection of TRX (100 µM); and femur fracture with a high dose of TRX (200 µM). TRX was administered intramuscularly every other day after surgery. X-rays radiography was taken to determine the status of fracture healing at week 4 and 6. At week 4 and 6 after surgery, animals were randomly selected to sacrifice and the femurs were collected for further analyses.

### Micro-Computer Tomography Scanning

The fractured femur was examined using a high-resolution micro-CT instrument (SkyScan1172, Bruker, German). The samples were scanned by the procedure with a source voltage of 70 keV, current of 80 µA, and 9 µm isotropic resolution. The region of interest (ROI) for scanning was set at 2.2 mm up and below of the fracture plate. Two-dimensional data obtained from the scanned femur were applied for the three-dimensional reconstruction and several morphometric parameters including trabecular bone volume (BV), tissue volume (TV), Bone surface (BS), BV/TV and BS/TV were calculated using the CT-VOX software (CtAN, Bruker, German).

### Bone Histomorphometry and Immunohistochemistry Staining

All femurs were initially fixed with 10% formalin for 72 h, followed by decalcification in 10% EDTA solution for 2 weeks. The samples were cut into 5 µm thick sections and stained with hematoxylin and eosin (H and E) according to the previous study (Molvik and Khan, 2015). The femurs were sliced along the long axis in the coronal plane for the bone-fracture study. IHC staining was performed as previously reported (Sun et al., 2015). Secretions were incubated with primary antibodies of osteocalcin (OCN, 1:100, abcam13418), osterix (OSX, 1:100, ab22552) and β-catenin (1:100; Cell Signaling Technology) overnight at 4°C. The horse-radish peroxidase-streptavidin system (Dako, United States) was applied for IHC signal detection, followed by counterstaining with hematoxylin. Finally, the images were captured under the Positive Fluorescence Microscope (Olympus BX53F, Japan). The analysis of the positive-stained cell area was performed using NIH ImageJ software.

### Data Analyses

At least triplicates were taken in each experiment and the data were recorded as the mean ± SD. Student’s t test and One-way ANOVA were used for statistically analysis between inter-groups analyses, and a *p*-value of less than 0.05 was considered statistically significant.

## Results

### TRX Exhibited No Significant Inhibitory Effects on Cell Viability of Human MSCs

**FIGURE 1:**
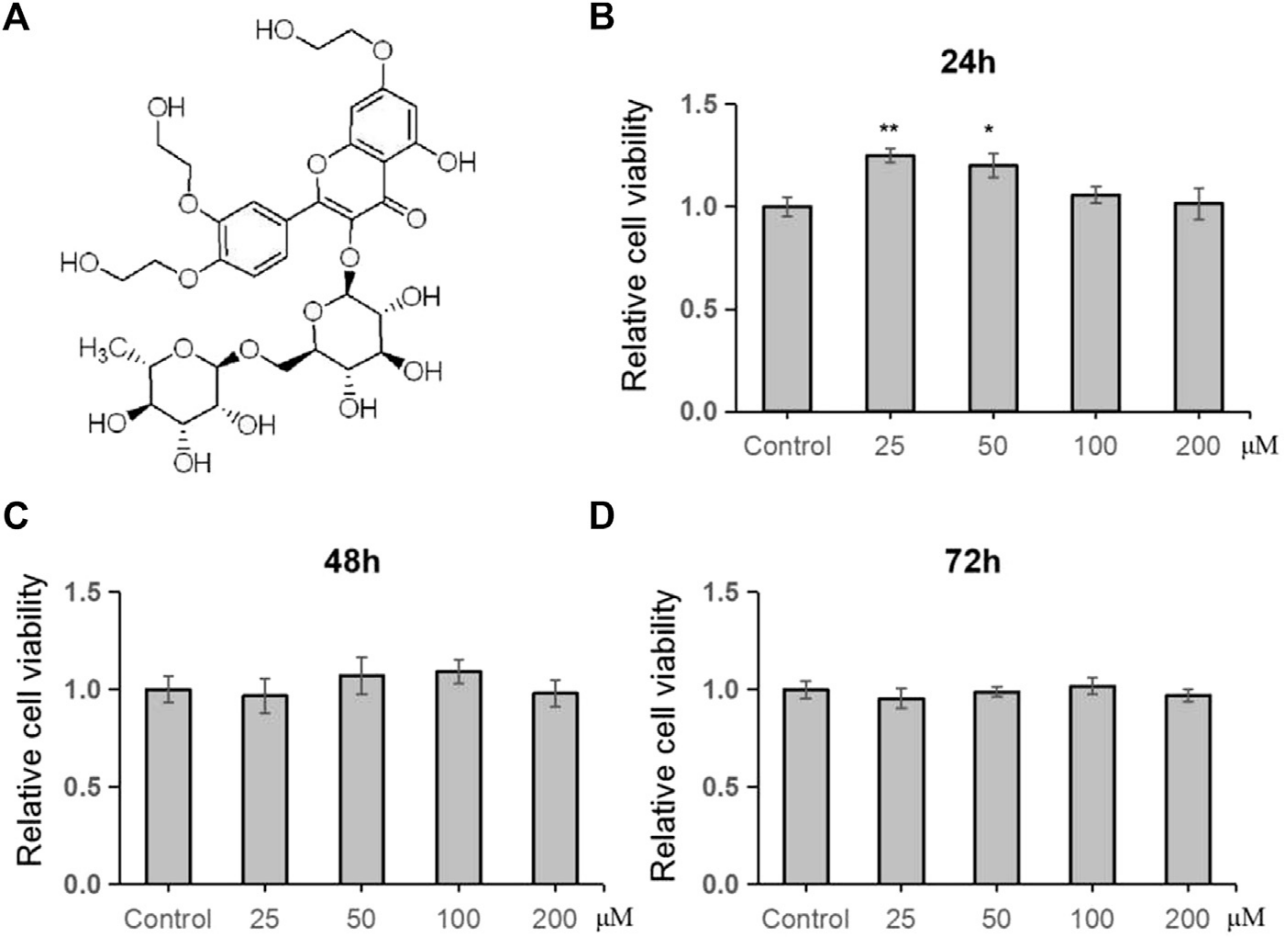
TRX had no significant effect on cell viability of MSCs. **(A)** The chemical structure of TRX. **(B–D)** The cell viability of TRX was examined by MTT after treating with TRX for 24, 48, and 72 h *, *p* < 0.05; **, *p* < 0.01; vs. Control.

In this study, we firstly examined the inhibitory effects of TRX on cell proliferation of human MSCs. By using MTT examination, TRX, its chemical structure showed in Figure 1A, exhibited no significant inhibitory effects on cell viability of human MSCs, even with the concentration of 200 µM (Figures 1B–D), suggesting it has low cytotoxicity to MSCs.

### TRX Promoted Osteogenic Differentiation of MSCs

**FIGURE 2:**
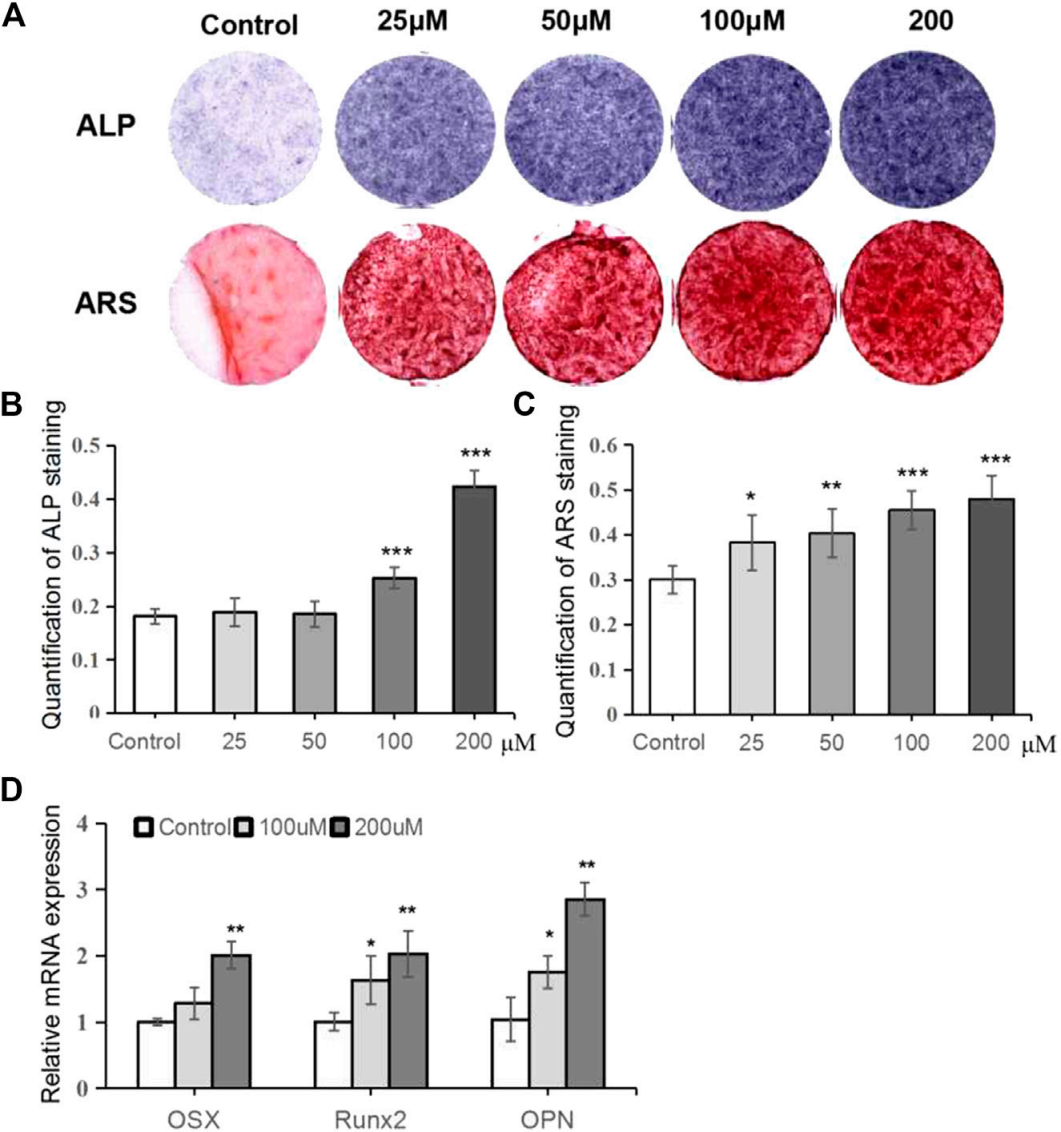
TRX promoted osteoblast differentiation of MSCs. **(A)** Qualitative examiation of ALP staining and ARS staining. **(B)** The quantitative assay of ALP activity. **(C)** The quantitative assay of ARS staining. **(D)** The quantitative assay of OD value. **(E)** The expression of several osteogenic marker genes was measured by qRT-PCR assays at day 14. *, *p* < 0.05; **, *p* < 0.01; ***, *p* < 0.001; vs. Control.

To investigate the effects of TRX on osteogenesis, TRX-treated MSCs were induced to differentiate into osteoblast. ALP activity, an early marker of osteogenic differentiation, was monitored at day 7. As shown in Figure 2A (up-panel) and 2B, TRX treatment enhanced the ALP activity in a dose-dependent manner from 0 to 200 µM. The ARS staining examination also confirmed that TRX promoted calcium nodule formation at day 14, consistent with ALP activity (Figure 2A down-panel and 2C). Based on the results of ALP activity and ARS staining, TRX with the concentration of 100 and 200 µM were selected for further investigation. We next examined the expression of osteogenic markers, including OSX, Runx2, and OPN by qRT-PCR examination; and the results showed that they were significantly up-regulated by 100 and 200 µM TRX, especially by 200 µM TRX (Figure 2D).

### TRX Induced the Activation of Wnt/β-Catenin Signaling in Human MSCs

**FIGURE 3:**
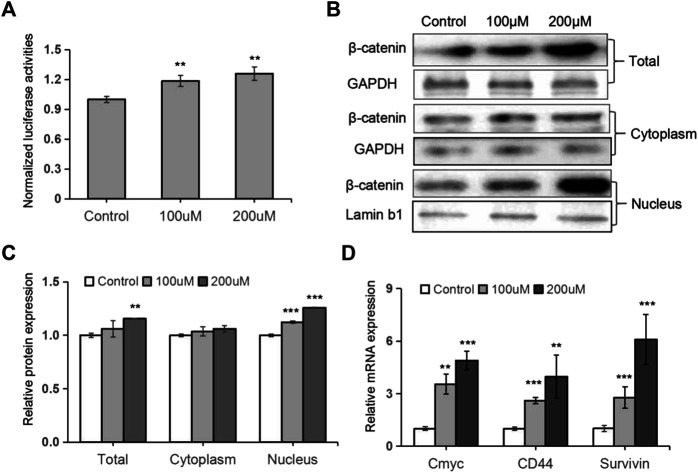
TRX induced the activation of Wnt/β-catenin signaling. **(A)** The normalized luciferase activity of TOPflash. **(B)** The expression of β-catenin in TRX-treated MSCss were examined by Western blotting. **(C)** Quantitative analyses of β-catenin expression. **(D)** The expression of several downstream targets of Wnt/β-catenin pathway were examined by qRT-PCR assays. *, *p* < 0.05; **, *p* < 0.01; ***, *p* < 0.001; vs. Control.

Considering that Wnt/β-catenin signaling plays a pivotal role in regulating osteoblast differentiation and bone formation, we wondered whether this signaling was involved in TRX-mediated osteogenesis. As shown in Figure 3A, the luciferase activities of the Wnt/β-catenin signaling reporter TOPflash were significantly promoted by TRX. And the expression of total β-catenin, the key regulator of Wnt signaling, was promoted by TRX treatment at protein level (Figure 3B). It is well known that β-catenin accumulates in the nucleus and stimulates Wnt/β-catenin signaling and regulates gene transcription. We therefore examined the expression of intranuclear β-catenin and intracytoplasmic β-catenin, and the results showed that intranuclear β-catenin were significantly increased in TRX treated MSC cells (Figures 3B,C). Furthermore, several downstream target genes of Wnt/β-catenin signaling such as Cmyc, CD44, and Survivin were examined, and they were obviously up-regulated by TRX (Figure 2D). All of these data suggest that TRX induced the activation of Wnt/β-catenin signaling during the osteogenic differentiation *via* stimulating the translocation of β-catenin from the cytoplasm to the nucleus.

### TRX Accelerated Bone Fracture Healing *in vivo*

**FIGURE 4:**
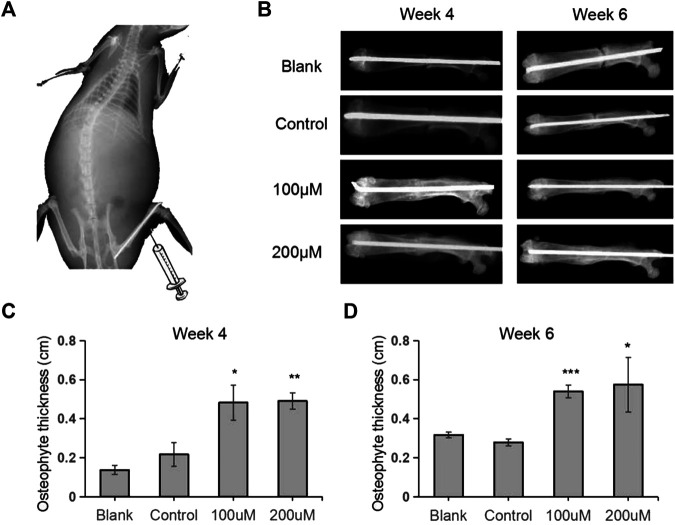
Quantitative analyses of osteophyte thickness by X-ray radiography. **(A)** The diagram of the femoral fracture model in rat. **(B)** X-ray images were taken during the fracture healing processes at week 4 and week 6. **(C–D)** The osteophyte thickness of the newly formed bone was analyzed at week 4 **(C)** and week 6 **(D)**. n = 6; *, *p* < 0.05; vs. Control.

To further evaluate whether API could enhance fracture healing *in vivo*, a rat fracture model was established and TRX were locally injected into the fracture sites every other day (Figure 4A). The broken bones were monitored by X-rays examination and the results showed that the gaps between the fracture sites almost disappeared in TRX treated groups while it also remained clearly visible in control groups, especially in the 200 µM TRX treated groups (Figure 4B). We also statistically evaluated the callus and the results showed that the thickness of newly formed callus was significantly improved by TRX administration at week 4 and 6 (Figures 4C,D). These data indicated that the fracture healing process was accelerated in TRX treated animals.

### TRX Promoted the New Bone Formation *in vivo*

**FIGURE 5:**
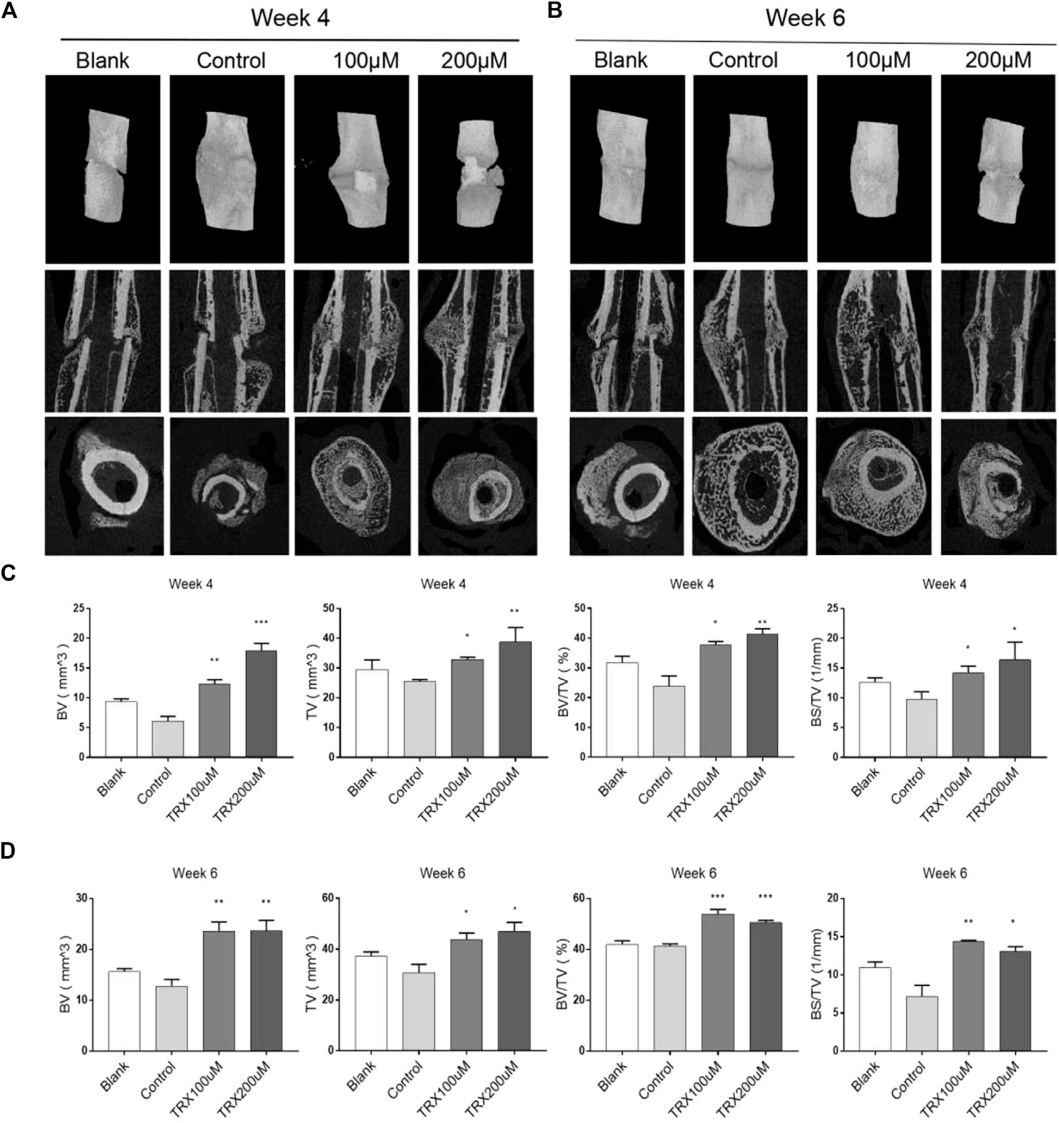
TRX improved the quality of new callus by micro-CT examination. **(A–B)**, Micro-CT examination of the femur fractured zone after 4 and 6 weeks treatment of TRX. **(C–D)**, The statistical diagram of BV, TV, BV/TV, and BS/TV. *, *p* < 0.05; **, *p* < 0.01; ***, *p* < 0.001; vs. Control.

**FIGURE 6:**
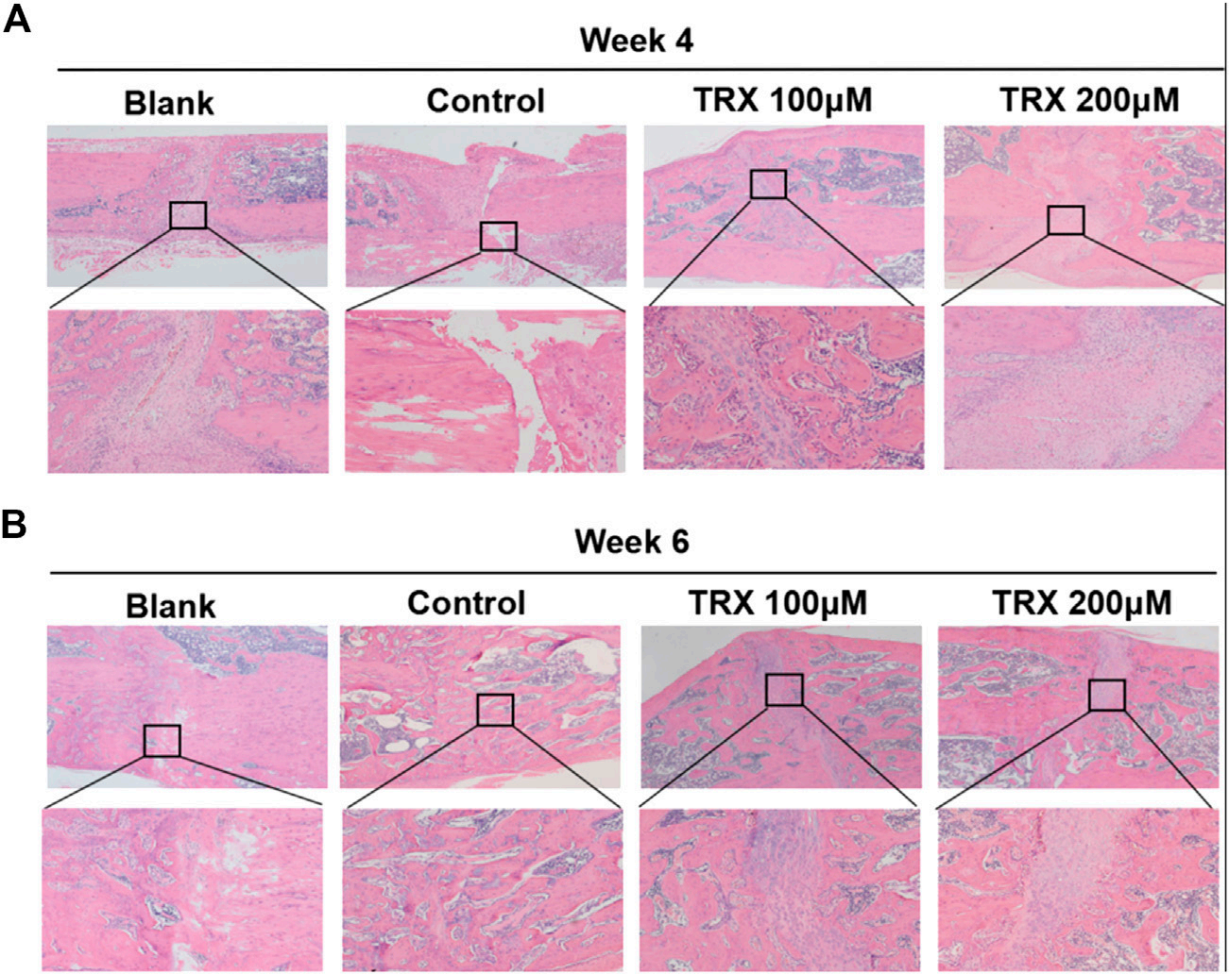
TRX promoted fracture healing by H and E staining assays. HE staining assays for the fracture sites at week 4 **(A)** and week 6 **(B)**.

**FIGURE 7:**
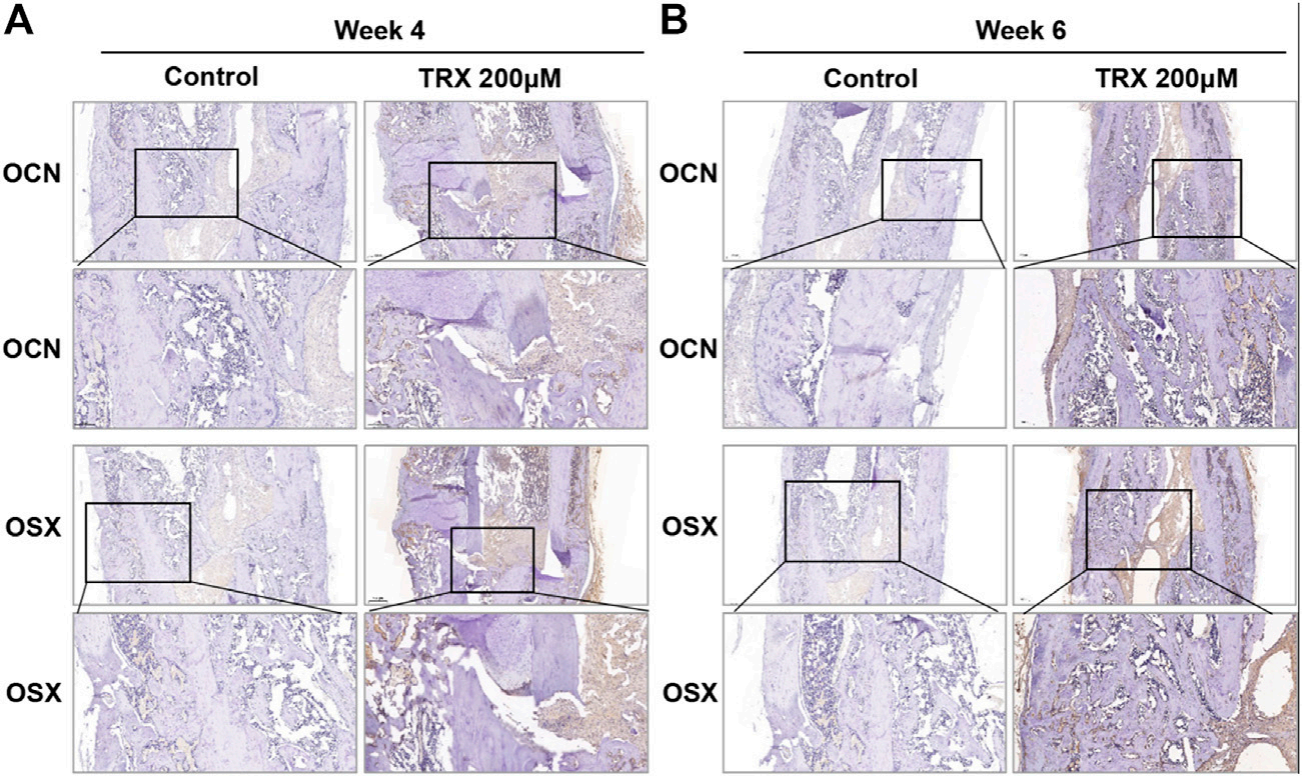
The expression of OSX and OCN was promoted in TRX-treated groups by immunohistochemistry (IHC) examination. The expression of OSX and OCN at the fracture sites was evaluated by IHC staining at week 4 **(A)** and week 6 **(B)**.

We next investigated the bone formation during the fracture sites using micro-CT scanning, and images were reconstructed by three-dimensional modeling system. As shown in Figures 5A,B, more mineralized calluses were observed in the TRX-treated groups when compared with their respective control groups at week 4 and 6. In addition, the bone volume (BV), tissue volume (TV), and bone surface (BS) were recorded, and the data showed that they were significantly increased by TRX treatment at week 4 and 6 (Figures 5C,D). The ratios of BV/TV and BS/TV also exhibited a significant increase in the TRX-treated groups at week 4 and 6 (Figures 5C,D), indicating more newly formed bone with TRX treatment. We further evaluated the newly formed bone tissues by histological examination, the H and E staining showed that more osteoblasts were converged at the fracture sites of TRX treatment groups, suggesting more active bone regeneration in TRX groups (Figures 6A,B). Moreover, the OCN and OSX expression in the animal tissues was evaluated by immunohistochemistry staining. Representative micrograph images showed that positive staining of OCN and OSX was increased in TRX-treated groups (Figures 7A,B, Supplementary Figure S1), indicating the promoting effect of TRX on bone formation. We also investigated the β-catenin expression around the fracture sites, and the results showed that it was promoted by TRX in animal specimens (Supplementary Figure S2).

## Discussion

Delayed healing and nonunion of fracture are the common orthopaedic diseases, which bring enormous burdens to patients and health care systems (Kostenuik and Mirza, 2017). Although a variety of approaches have been discovered to promote fracture healing, there are currently no approved pharmacological agents for the treatment of established nonunions and delayed healing of fracture (Bigham-Sadegh and Oryan, 2015; An et al., 2016). Series of natural products derived from traditional Chinese medicine (TCM) have demonstrated to benefit fracture healing, which brings a new sight for the treatment (Zhang et al., 2009; Piao et al., 2019). In the present study, TRX, a kind of natural flavone which derived from various fruits and vegetables, was found to promote osteogenesis *in vitro* and accelerate bone formation *in vivo*, suggesting that it may be a promising activator to bone repair.

As a derivative of the natural bioflavonoid rutin, TRX has been reported to have anti-oxidant, anti-inflammatory and anti-cancer properties (Horcajadamolteni et al., 2010; Lu et al., 2011). Although there is no study on the effects of TRX in musculskeletal system, its precursor rutin was reported to have a protective role against bone loss (Xiao, et al., 2019). In the present study, TRX was found to promote osteogenic differentiation of MSCs by ALP activities, calcium nodule formation and osteogenic marker genes expression. On the other hand, the cytotoxicity of TRX is a question worthy of our concern because serious cytotoxicity could inhibit its clinical application. Our study showed that TRX, even with the concentration of 200 µM, has mild inhibitory effect on MSCs viability. The striking osteogenic effects combined with low cytotoxicity make TRX to be a promising strategy for fracture patients.

As well known, Wnt/β-catenin signaling is crucial for embryonic development and tissue homeostasis (Aline et al., 2013). A number of evidence have demonstrated that this signaling plays key regulatory roles in cellular differentiation and matrix formation during skeletal development and formation (Chen et al., 2007; Aline et al., 2013). The activation of canonical Wnt/β-catenin signaling cascade is significant for the bone formation and regeneration (Duan and Bonewald, 2016; Tian et al., 2018); and the agonist of this signaling has been proved to accelerate the bone repair in the early stage of fracture healing (Hong, et al., 2019). In this study, we found that the expression of intranuclear β-catenin and several downstream target genes of Wnt/β-catenin was up-regulated by TRX, indicating the activation of Wnt/β-catenin signaling in TRX-mediated osteogenesis.

To further verify the *in vivo* effect of TRX, an established femoral fracture model was applied (Nyman et al., 2009; Liang et al., 2019). 12-week-old (3 months) adult rats were selected to establish this fracture model because the animals of this age have been mature, and stopped growing and developing. Once the fracture happened, it needs a long time to self-healing which facilitate to evaluate the TRX’s effects. In our study, TRX was locally injected into fracture sites to stimulate the healing process. The results showed that TRX administration could promote the new bone formation and stimulate the fracture healing based on the X-ray examination, micro-CT scanning, and HE staining. More specially, we observed more blood vessels in the fracture zones of TRX-treated groups. Angiogenesis, the formation and remodeling of new blood vessels, plays a pivotal role in bone development (Sivaraj and Adams, 2016; Diomede et al., 2020). As is widely reported, osteogenesis is coupled with angiogenesis during bone regeneration and formation (Carano and Filvaroff, 2003; Kusumbe et al., 2014). Therefore, new blood vessel formation is essential during both primary bone development as well as fracture repair in adults^35^. In the HE staining images, we observed many like-blood vessels in the TRX-treated tissues. We therefore hypothesized that TRX accelerated bone fracture healing through promoting osteogenesis and stimulating blood vessel formation. The function of TRX on angiogenesis will be deeply examined in near future.

In summary, our results demonstrated that TRX could promote osteogenic differentiation *in vitro* and accelerate fracture healing *in vivo.* And the underlying mechanism investigation showed that the activated Wnt/β-catenin signaling involved in the TRX-mediated bone regeneration. TRX thereby may be considered as a potential agonist of Wnt/β-catenin signaling to develop the bone-protective therapeutic strategy for the clinical practice.

## Data Availability

The original contributions presented in the study are included in the article/Supplementary Material, further inquiries can be directed to the corresponding authors.

## References

[B1] Aline H. Thorsten S. Ronny B. Tim W. Anna R. Melanie H. L. (2013). The Wnt Serpentine Receptor Frizzled-9 Regulates New Bone Formation in Fracture Healing. Plos One 8 (12), e84232. 10.1371/journal.pone.0084232 24391920 PMC3877253

[B2] An J. Yang H. Zhang Q. Liu C. Zhao J. Zhang L. (2016). Natural Products for Treatment of Osteoporosis: the Effects and Mechanisms on Promoting Osteoblast-Mediated Bone Formation. Life Sci. 147, 46–58. 10.1016/j.lfs.2016.01.024 26796578

[B3] Bigham-Sadegh A. Oryan A. (2015). Basic Concepts Regarding Fracture Healing and the Current Options and Future Directions in Managing Bone Fractures. Int. Wound J. 12 (3), 238–247. 10.1111/iwj.12231 24618334 PMC7950494

[B4] Carano R. A. D. Filvaroff E. H. (2003). Angiogenesis and Bone Repair. Drug Discov. Today 8 (21), 980–989. 10.1016/s1359-6446(03)02866-6 14643161

[B5] Chamberlain G. Fox J. Ashton B. Middleton J. (2007). Concise Review: Mesenchymal Stem Cells: Their Phenotype, Differentiation Capacity, Immunological Features, and Potential for Homing. Stem Cells 25, 2739–2749. 10.1634/stemcells.2007-0197 17656645

[B6] Chen Y. Whetstone H. C. Lin A. C. Nadesan P. Wei Q. Poon R. (2007). Beta-catenin Signaling Plays a Disparate Role in Different Phases of Fracture Repair: Implications for Therapy to Improve Bone Healing. Plos Med. 4 (7), e249. 10.1371/journal.pmed.0040249 17676991 PMC1950214

[B7] Diomede F. Marconi G. D. Fonticoli L. Pizzicanella J. Merciaro I. Bramanti P. (2020). Functional Relationship between Osteogenesis and Angiogenesis in Tissue Regeneration. Int. J. Mol. Sci. 21, 3242. 10.3390/ijms21093242 PMC7247346 32375269

[B8] Duan P. Bonewald L. F. (2016). The Role of the Wnt/β-Catenin Signaling Pathway in Formation and Maintenance of Bone and Teeth. Int. J. Biochem. Cel Biol. 77 (Pt A), 23–29. 10.1016/j.biocel.2016.05.015 PMC4958569 27210503

[B9] Farajdokht F. Amani M. Mirzaei Bavil F. Alihemmati A. Mohaddes G. Babri S. (2017). Troxerutin Protects Hippocampal Neurons Against Amyloid Beta-Induced Oxidative Stress and Apoptosis. Excli J. 16, 1081–1089. 10.17179/excli2017-526 29285004 PMC5735350

[B10] Feng L. Shi L. Lu Y.-F. Wang B. Tang T. Fu W.-M. (2018). Linc-ROR Promotes Osteogenic Differentiation of Mesenchymal Stem Cells by Functioning as a Competing Endogenous RNA for miR-138 and miR-145. Mol. Ther. - Nucleic Acids 11, 345–353. 10.1016/j.omtn.2018.03.004 29858070 PMC5992460

[B11] Foulke B. A. Kendal A. R. Murray D. W. Pandit H. (2016). Fracture Healing in the Elderly: A Review. Maturitas 92, 49–55. 10.1016/j.maturitas.2016.07.014 27621238

[B12] Freires I. A. Santaella G. M. de Cássia Orlandi Sardi J. Rosalen P. L. (2017). The Alveolar Bone Protective Effects of Natural Products: A Systematic Review. Arch. Oral Biol. 87, 196–203. 10.1016/j.archoralbio.2017.12.019 29306777

[B13] Ghasroldasht M. M. Matin M. M. Mehrjerdi H. K. Naderi-Meshkin H. Moradi A. Rajabioun M. (2018). Application of Mesenchymal Stem Cells to Enhance Non-union Bone Fracture Healing. J. Biomed. Mater. Res. A 107, 301–311. 10.1002/jbm.a.36441 29673055

[B14] Hong G. He X. Shen Y. Chen X. Wei Q. (2019). Chrysosplenetin Promotes Osteoblastogenesis of Bone Marrow Stromal Cells via Wnt/β-Catenin Pathway and Enhances Osteogenesis in Estrogen Deficiency-Induced Bone Loss. Stem Cel Res. Ther. 10, 277. 10.1186/s13287-019-1375-x PMC6716882 31464653

[B15] Horcajada-Molteni M. N. Crespy V. Coxam V. Davicco M. J. Rémésy C. Barlet J. P. (2010). Rutin Inhibits Ovariectomy-Induced Osteopenia in Rats. J. Bone Miner Res. 15 (11), 2251–2258. 10.1359/jbmr.2000.15.11.2251 11092407

[B16] Kasper G. Mao L. Geissler S. Draycheva A. Trippens J. Kühnisch J. (2009). Insights into Mesenchymal Stem Cell Aging: Involvement of Antioxidant Defense and Actin Cytoskeleton. Stem Cells 27, 1288–1297. 10.1002/stem.49 19492299

[B17] Kostenuik P. Mirza F. M. (2017). Fracture Healing Physiology and the Quest for Therapies for Delayed Healing and Nonunion. J. Orthop. Res. 35, 213–223. 10.1002/jor.23460 27743449 PMC6120140

[B18] Kusumbe A. P. Ramasamy S. K. Adams R. H. (2014). Coupling of Angiogenesis and Osteogenesis by a Specific Vessel Subtype in Bone. Nature 507 (7492), 323–328. 10.1038/nature13145 24646994 PMC4943525

[B19] Liang W. C. Wong C. W. Liang P. P. Shi M. Cao Y. Rao S. T. (2019). Translation of the Circular RNA Circβ-Catenin Promotes Liver Cancer Cell Growth Through Activation of the Wnt Pathway. Genome Biol. 20, 84. 10.1186/s13059-019-1685-4 31027518 PMC6486691

[B20] Lu J. Wu D.-M. Zheng Z.-H. Zheng Y.-L. Hu B. Zhang Z.-F. (2011). Troxerutin Protects against High Cholesterol-Induced Cognitive Deficits in Mice. Brain 134, 783–797. 10.1093/brain/awq376 21252113

[B21] Molvik H. Khan W. (2015). Bisphosphonates and Their Influence on Fracture Healing: a Systematic Review. Osteoporos. Int. 26 (4), 1251–1260. 10.1007/s00198-014-3007-8 25572046

[B22] Nyman J. S. Munoz S. Jadhav S. Mansour A. Yoshii T. Mundy G. R. (2009). Quantitative Measures of Femoral Fracture Repair in Rats Derived by Micro-computed Tomography. J. Biomech. 42, 891–897. 10.1016/j.jbiomech.2009.01.016 19281987

[B23] Piao H. Rong Y. Xue Z. Lei W. Xisong K. Qu Y. (2019). Activating Wnt/β-Catenin Signaling Pathway for Disease Therapy: Challenges and Opportunities. Pharmacol. Ther. 196, 79–90. 10.1016/j.pharmthera.2018.11.008 30468742

[B24] Praemer A. Furner S. Rice D. P. (1999). Musculoskeletal Conditions in the United States. Rosemont, Illinois, USA: Amer Acad Orthop Surg. Chapter1.

[B25] Secreto F. J. Hoeppner L. H. Westendorf J. J. (2009). Wnt Signaling During Fracture Repair. Curr. Osteoporos. Rep. 7 (2), 64–69. 10.1007/s11914-009-0012-5 19631031 PMC2972700

[B26] Shu L. Zhang W. Huang C. Huang G. Su G. (2017). Troxerutin Protects Against Myocardial Ischemia/Reperfusion Injury via Pi3k/Akt Pathway in Rats. Cell Physiol Biochem 44, 1939–1948. 10.1159/000485884 29241161

[B27] Sivaraj K. K. Adams R. H. (2016). Blood Vessel Formation and Function in Bone. Development 143, 2706–2715. 10.1159/000485884 27486231

[B28] Sun Y. Xu L. Huang S. Hou Y. Liu Y. Chan K.-M. (2015). Mir-21 Overexpressing Mesenchymal Stem Cells Accelerate Fracture Healing in a Rat Closed Femur Fracture Model. Biomed. Res. Int. 2015, 1–9. 10.1155/2015/412327 PMC4386680 25879024

[B29] Tian X. J. Zhou D. Fu H. Zhang R. Wang X. Huang S. (2018). Sequential Wnt Agonist Then Antagonist Treatment Accelerates Tissue Repair and Minimizes Fibrosis. iScience 23, 101047. 10.1016/j.isci.2020.101047 PMC7186527 32339988

[B30] Westgeest J. Weber D. Dulai S. K. Bergman J. W. Buckley R. Beaupre L. A. (2016). Factors Associated with Development of Nonunion or Delayed Healing After an Open Long Bone Fracture. J. Orthopaedic Trauma 30, 149–155. 10.1097/bot.0000000000000488 26544953

[B31] Xiao Y. Wei R. Yuan Z. Lan X. Kuang J. Hu D. (2019). Rutin Suppresses Fndc1 Expression in Bone Marrow Mesenchymal Stem Cells to Inhibit Postmenopausal Osteoporosis. Am. J. Transl Res. 11, 6680–6690. 31737218 PMC6834492

[B32] Yang Y.-T. MengHu J.-H. B. Hu B. Ma C.-Y. Zhao C.-C. Teng W.-S. -Y. (2018). A Novel Anti-osteoporotic Agent that Protects against Postmenopausal Bone Loss by Regulating Bone Formation and Bone Resorption. Life Sci. 209, 409–419. 10.1016/j.lfs.2018.08.014 30096387

[B33] Zamanian M. Bazmandegan G. Sureda A. Sobarzo-Sanchez E. Shirooie S. (2021). The Protective Roles and Molecular Mechanisms of Troxerutin (Vitamin P4) for Treatment of Chronic Diseases: A Mechanistic Review. Curr. Neuropharmacology 19, 97–110. 10.2174/1570159X18666200510020744 PMC7903491 32386493

[B34] Zhang J.-F. Fu W.-M. He M.-L. Wang H. Wang W.-M. Yu S.-C. (2011). Mir-637 Maintains the Balance Between Adipocytes and Osteoblasts by Directly Targeting Osterix. Mol. Biol. Cel 22 (21), 3955–3961. 10.1091/mbc.e11-04-0356 PMC3204058 21880893

[B35] Zhang J.-F. Li G. Chan C.-Y. Meng C.-L. Lin M. C.-M. Chen Y.-C. (2010). Flavonoids of Herba Epimedii Regulate Osteogenesis of Human Mesenchymal Stem Cells Through BMP and Wnt/β-Catenin Signaling Pathway. Mol. Cell Endocrinol. 314, 70–74. 10.1016/j.mce.2009.08.012 19703516

[B36] Zhang J. F. Li G. Meng C. L. Dong Q. Chan C. Y. He M. L. (2009). Total Flavonoids of Herba Epimedii Improves Osteogenesis and Inhibits Osteoclastogenesis of Human Mesenchymal Stem Cells. Phytomedicine 16 (6-7), 521–529. 10.1016/j.phymed.2009.01.003 19394806

