# Supplementary figures and images for: Troxerutin Stimulates Osteoblast Differentiation of Mesenchymal Stem Cell and Facilitates Bone Fracture Healing
Source: Front Pharmacol. 2021 Aug 9;12:723145. doi: 10.3389/fphar.2021.723145 (PMC8381475; doi:10.3389/fphar.2021.723145)

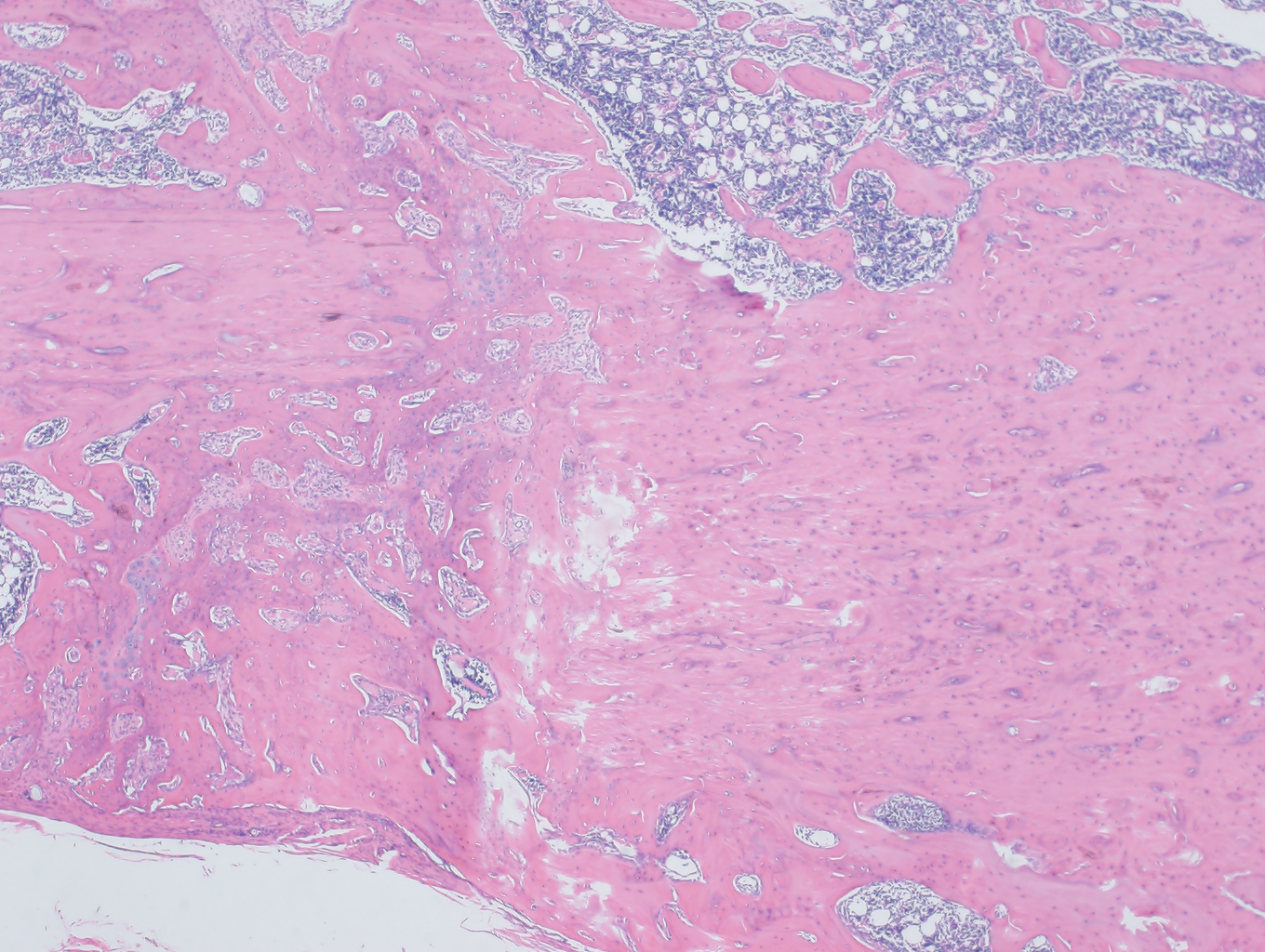

Supplement: Supplementary file 1 [file DataSheet3.ZIP › Figure 6-1 HE/Blank-6w-1.tif]

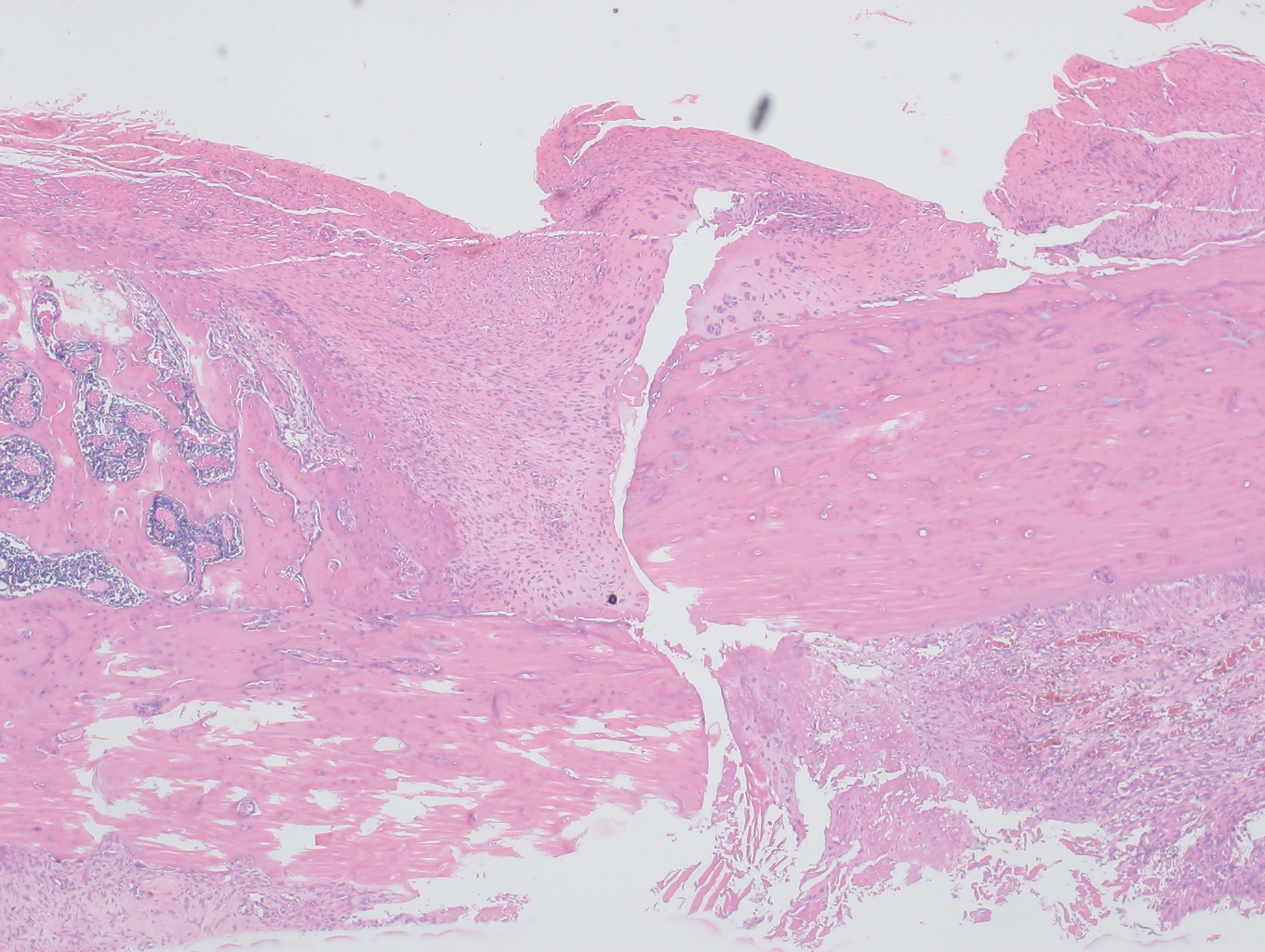

Supplement: Supplementary file 1 [file DataSheet3.ZIP › Figure 6-1 HE/Control-4w-1.tif]

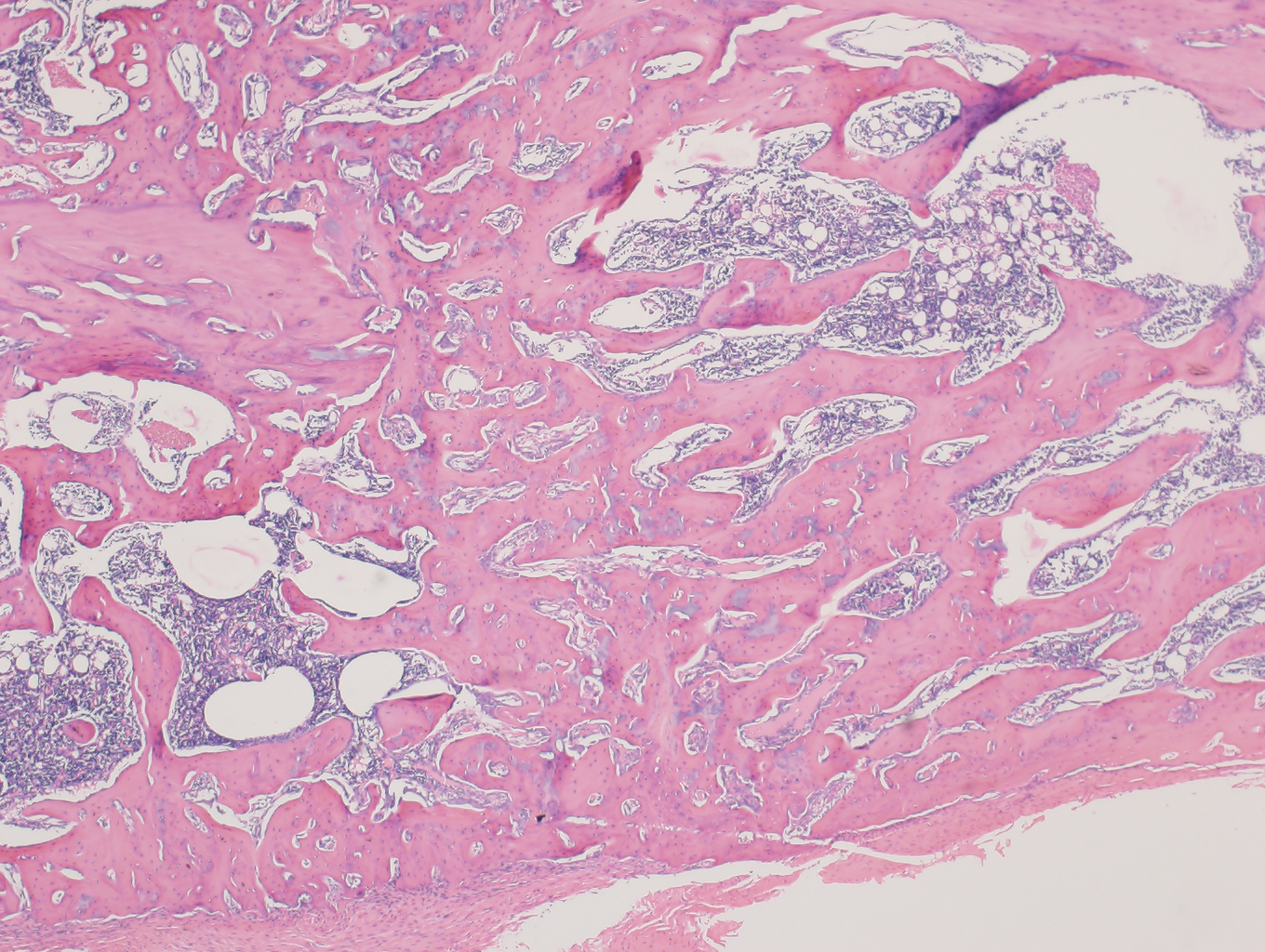

Supplement: Supplementary file 1 [file DataSheet3.ZIP › Figure 6-1 HE/Control-6w-1.tif]

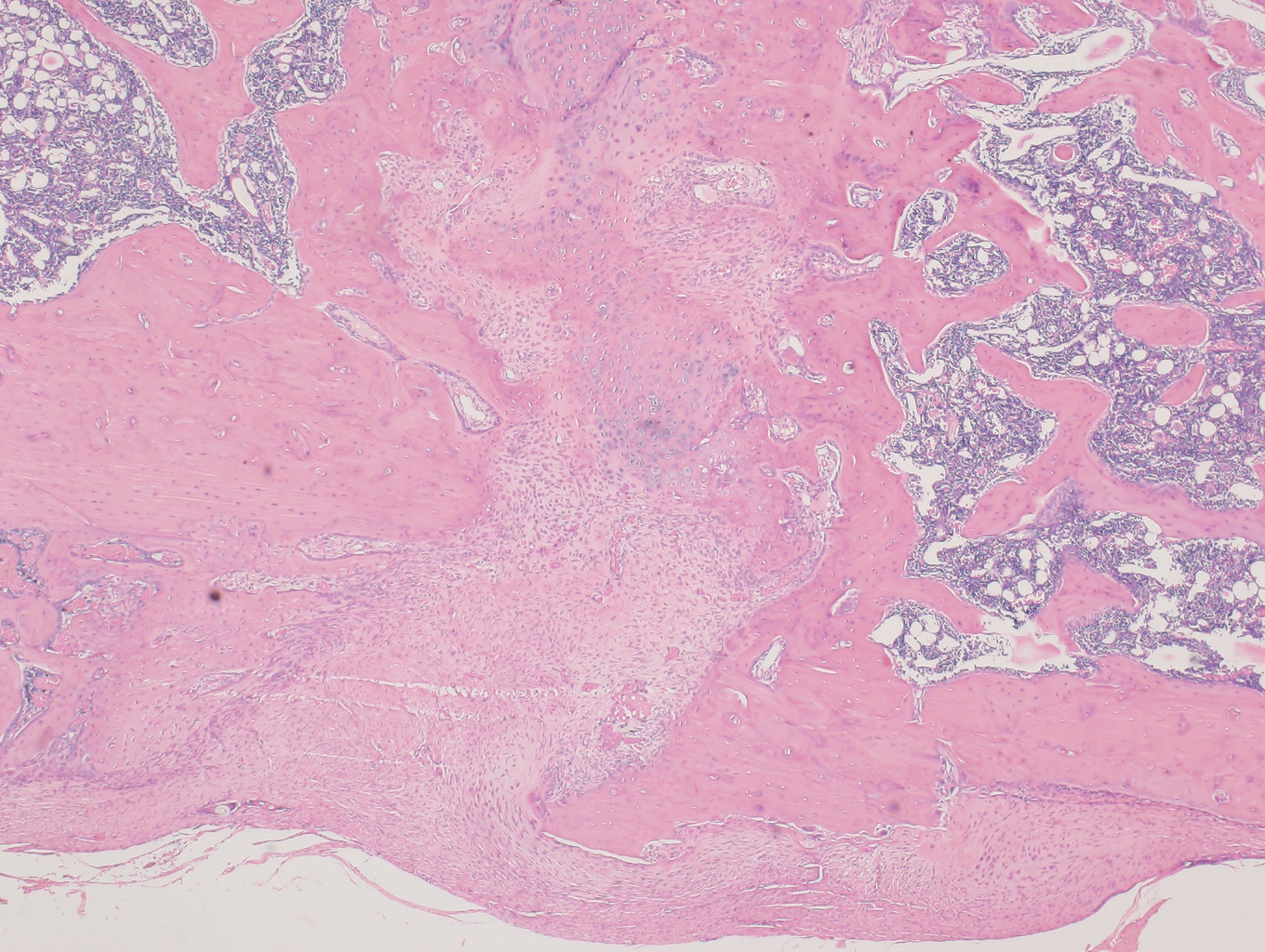

Supplement: Supplementary file 1 [file DataSheet3.ZIP › Figure 6-1 HE/TRX-200uM-4W1.tif]

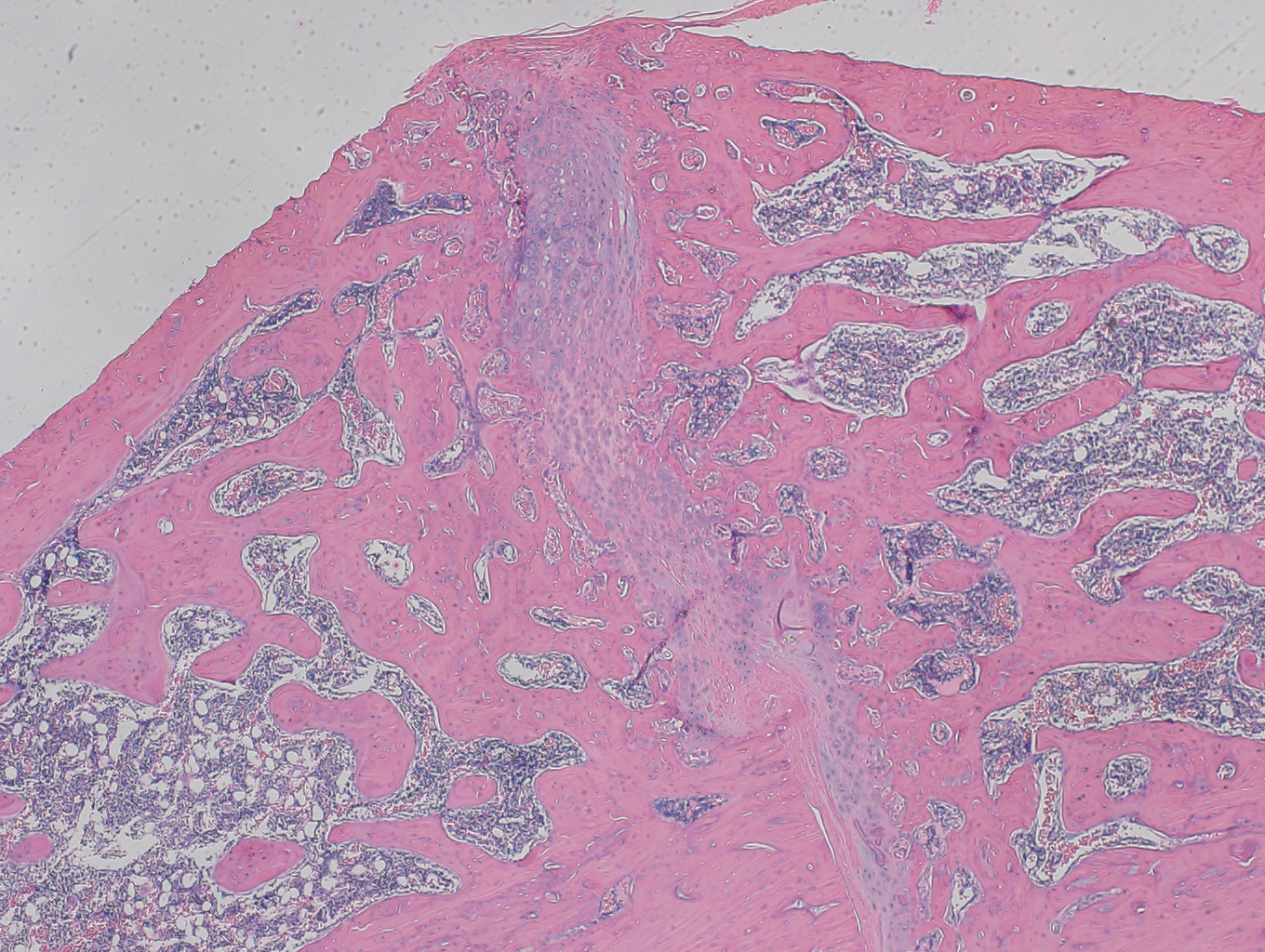

Supplement: Supplementary file 1 [file DataSheet3.ZIP › Figure 6-1 HE/TRX100uM- 6w -1.tif]

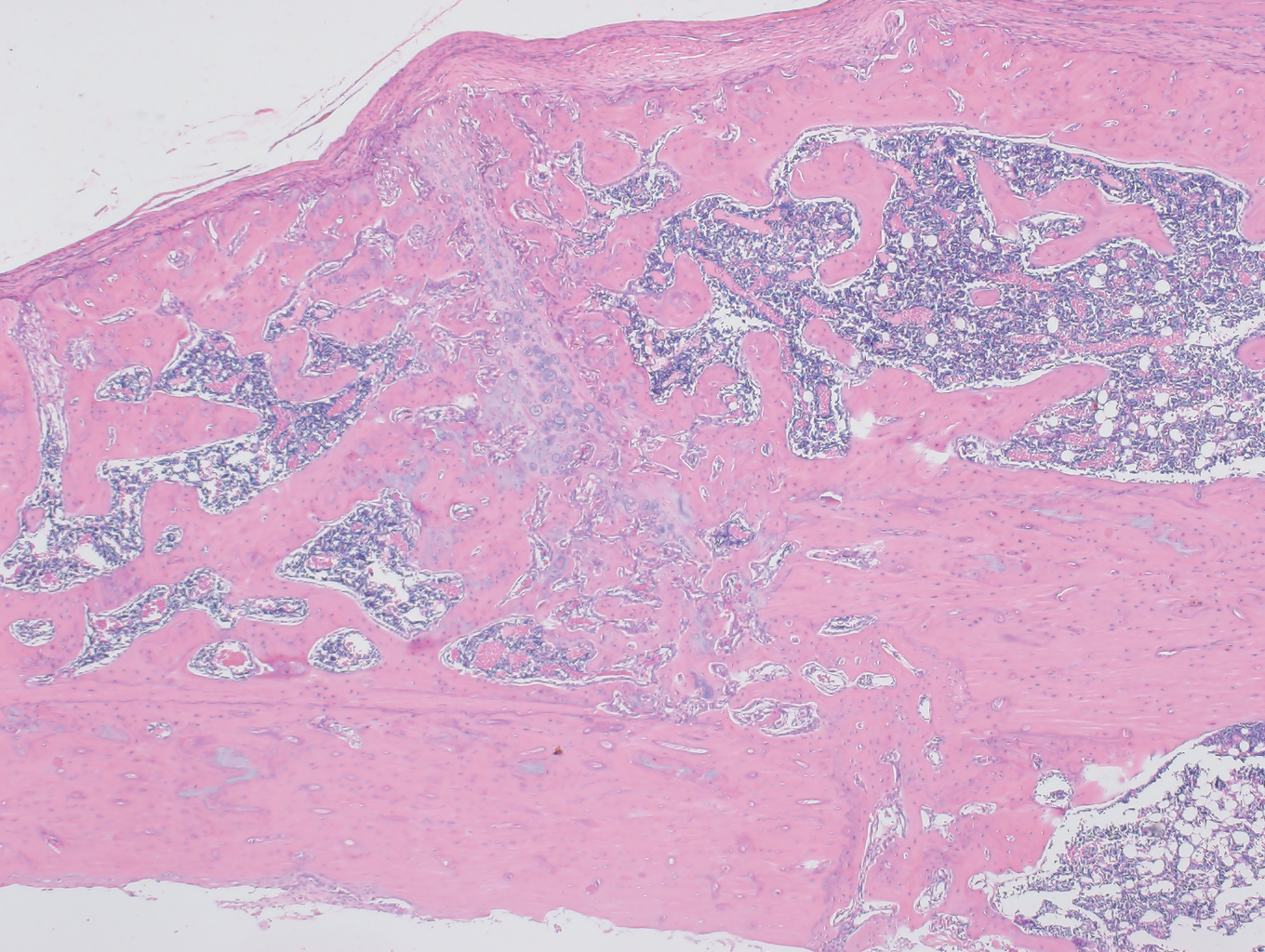

Supplement: Supplementary file 1 [file DataSheet3.ZIP › Figure 6-1 HE/TRX100uM-4w-1.tif]

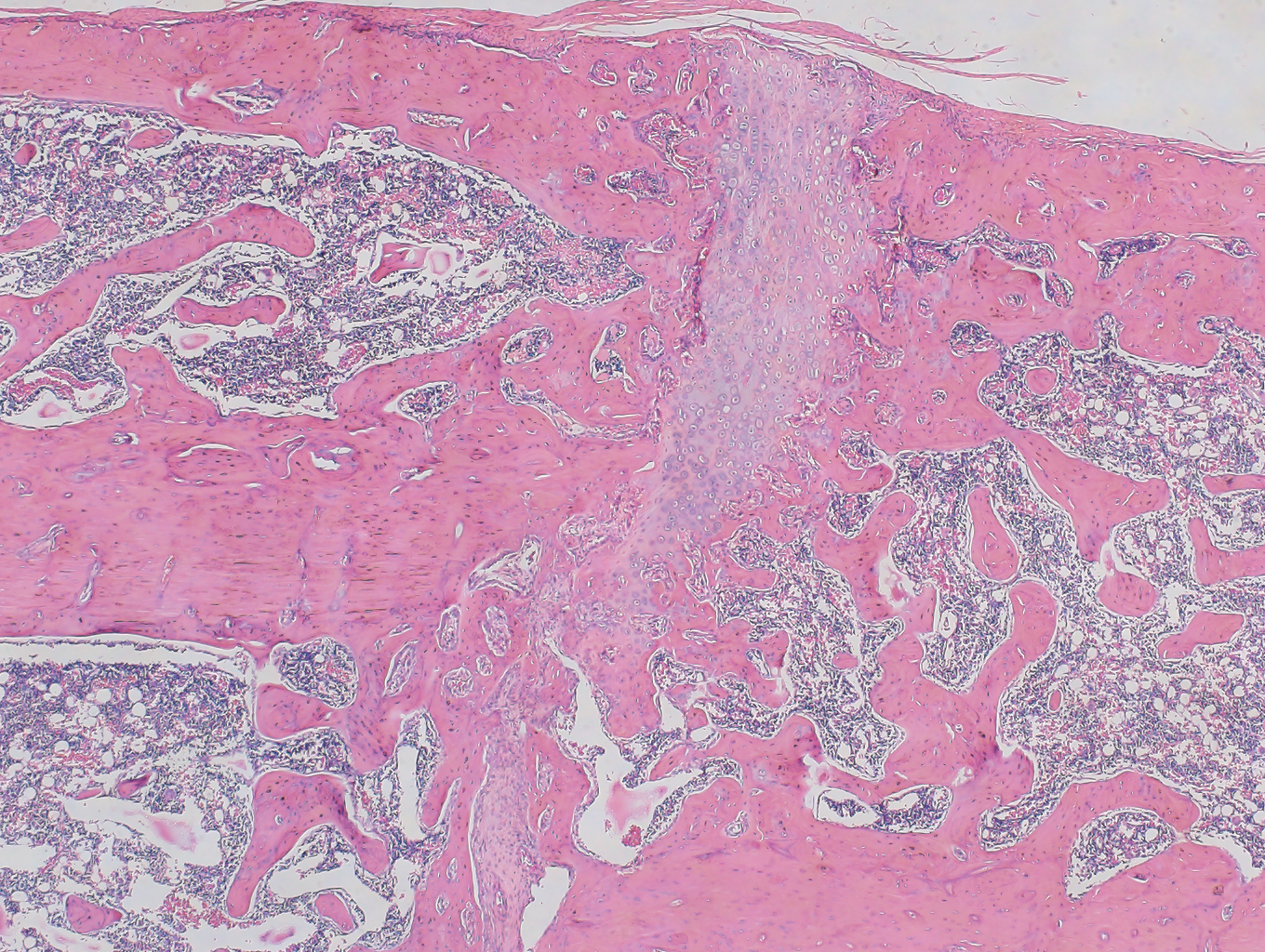

Supplement: Supplementary file 1 [file DataSheet3.ZIP › Figure 6-1 HE/TRX200uM-6W-1.tif]

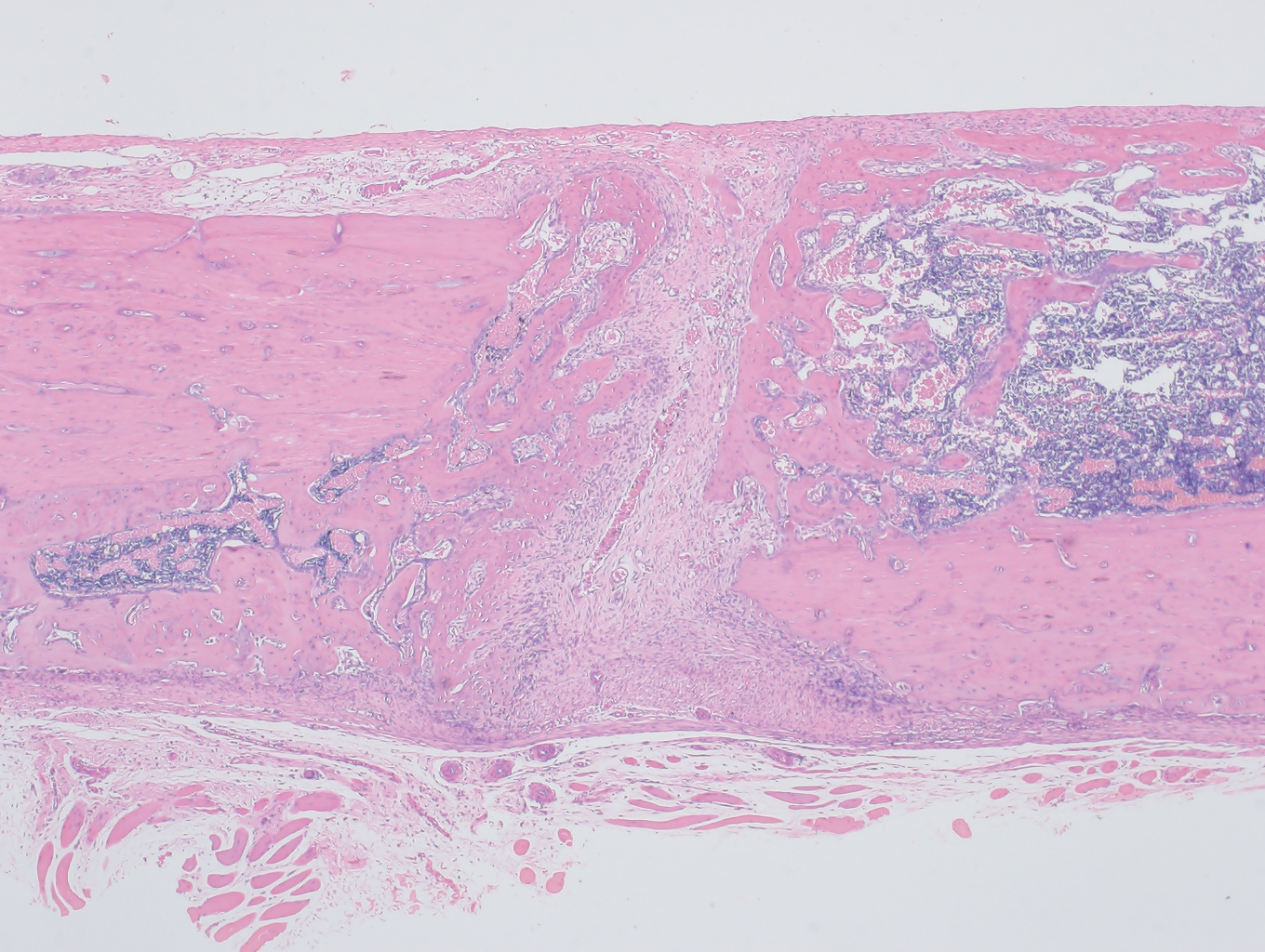

Supplement: Supplementary file 1 [file DataSheet3.ZIP › Figure 6-1 HE/blank 4w -1.tif]

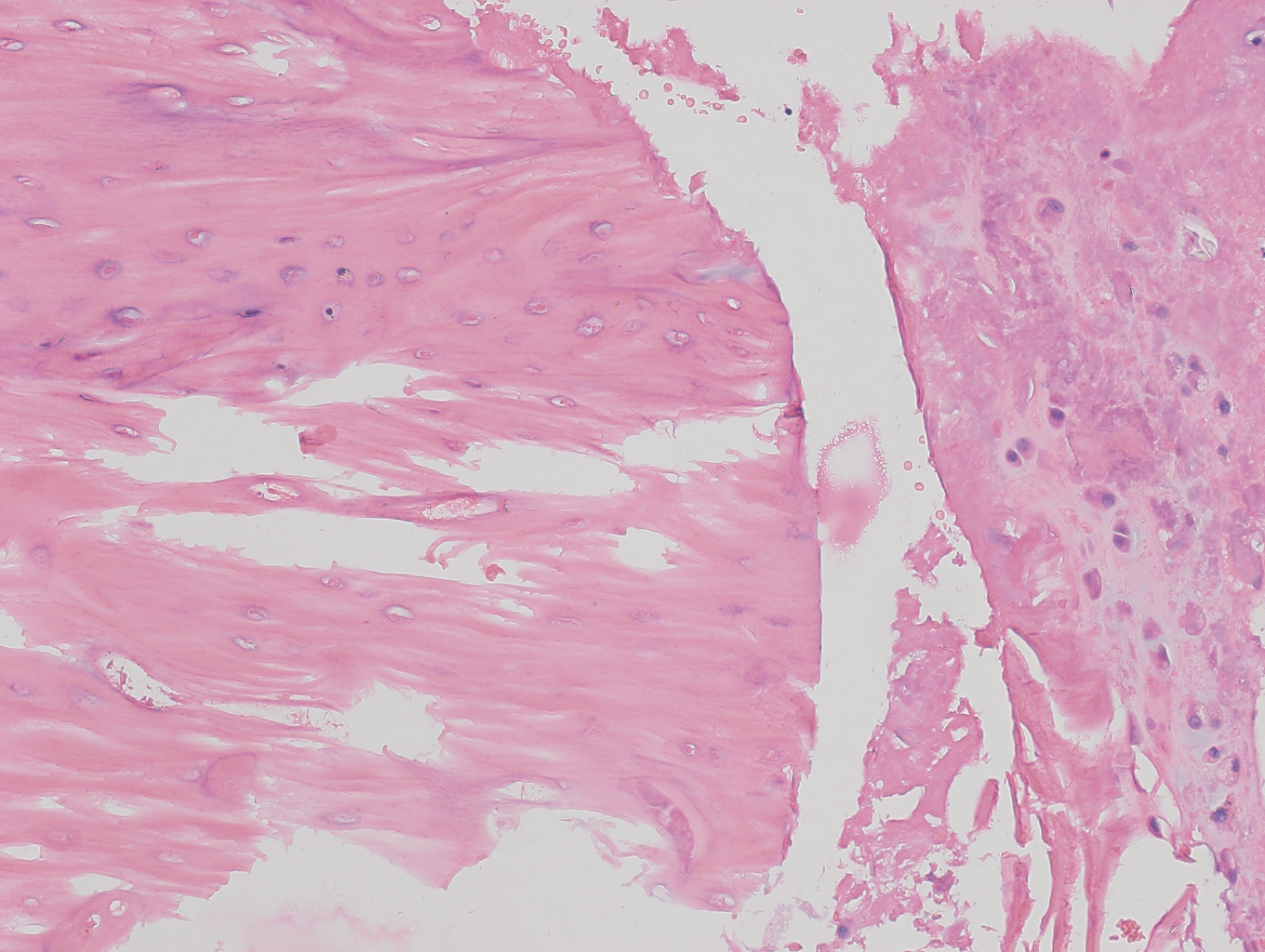

Supplement: Supplementary file 2 [file DataSheet4.ZIP › Figure 6-2 HE/Control-4w-2.tif]

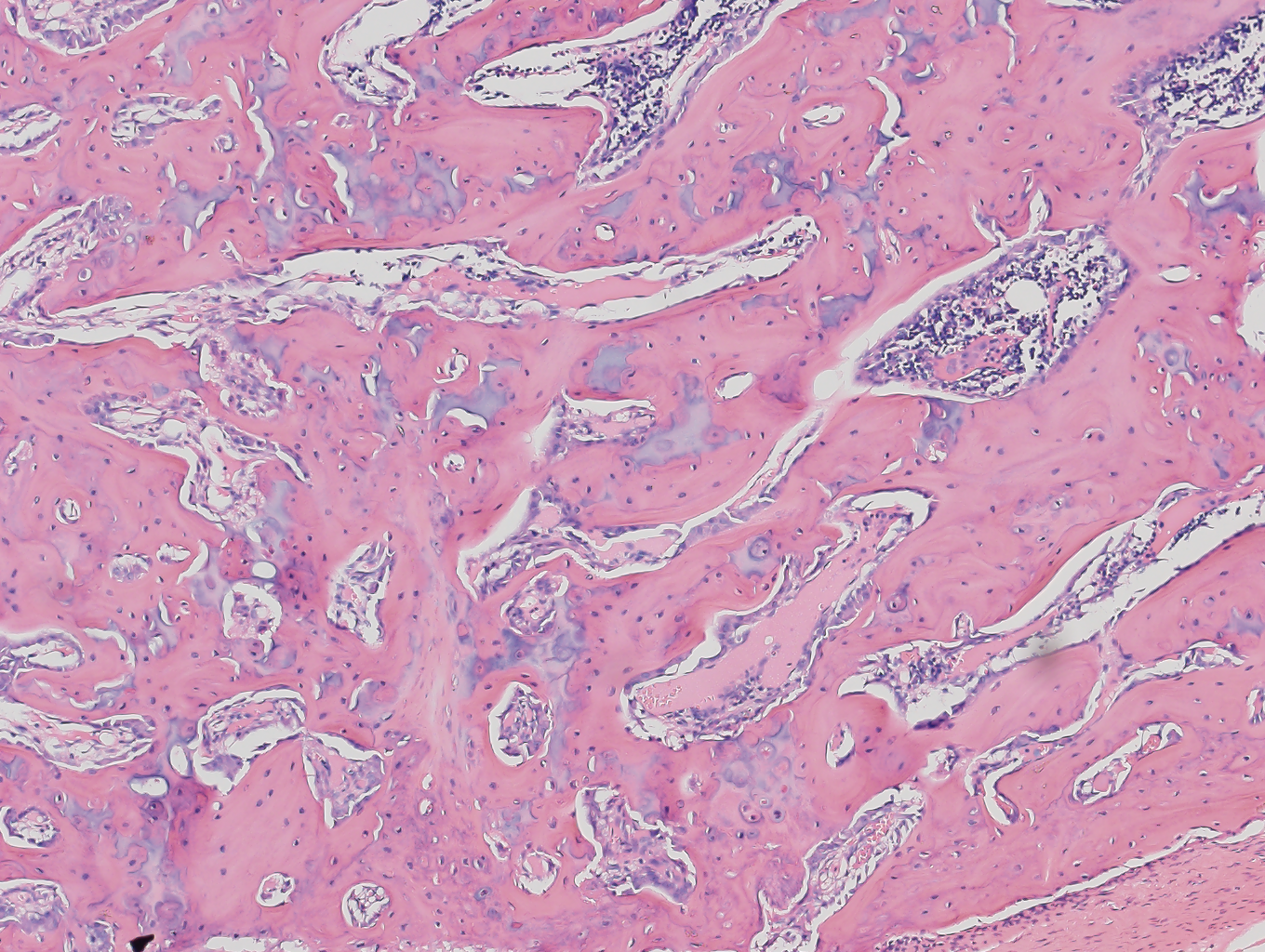

Supplement: Supplementary file 2 [file DataSheet4.ZIP › Figure 6-2 HE/Control-6w-2.tif]

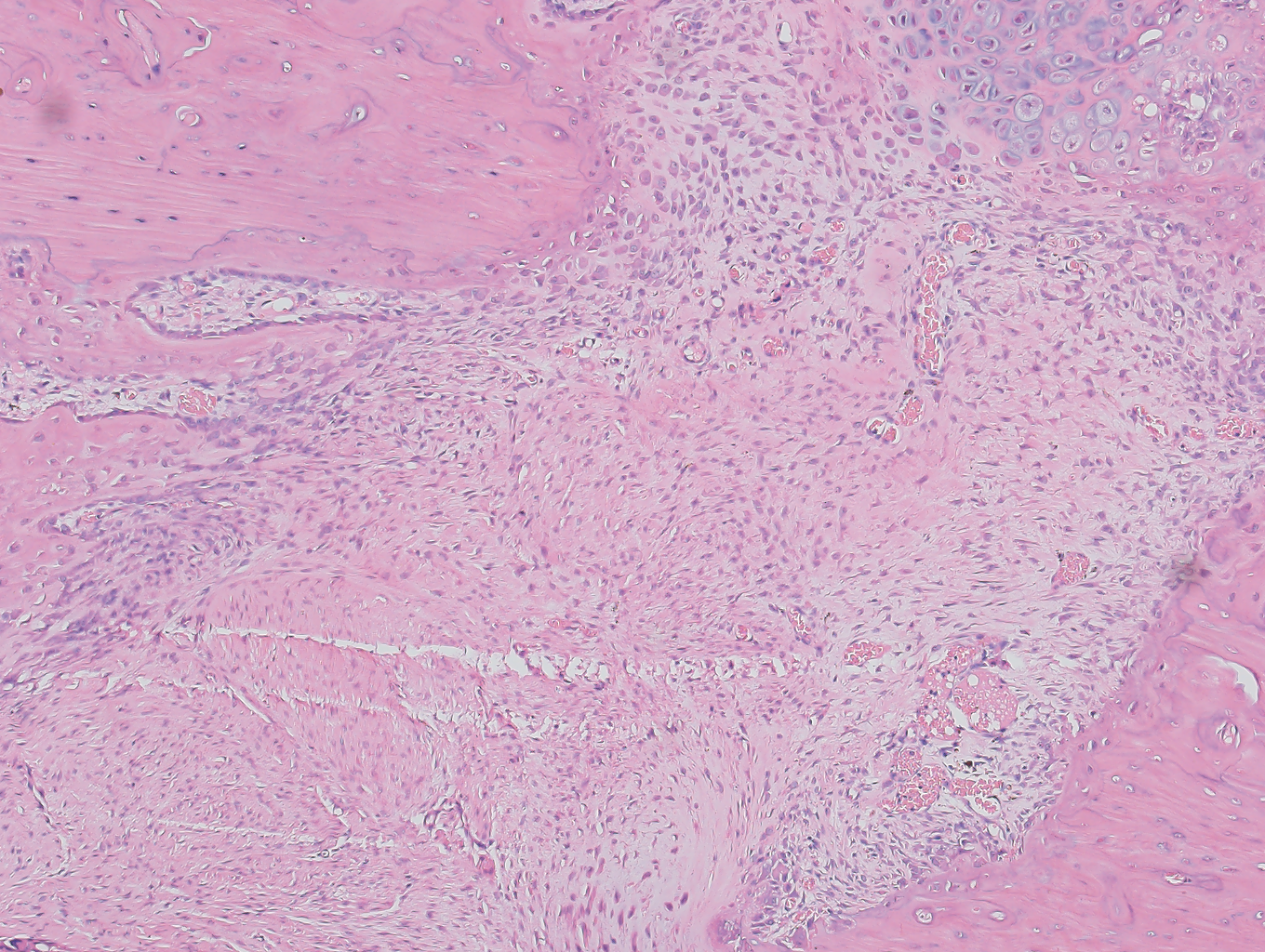

Supplement: Supplementary file 2 [file DataSheet4.ZIP › Figure 6-2 HE/TRX-200uM-4W-2.tif]

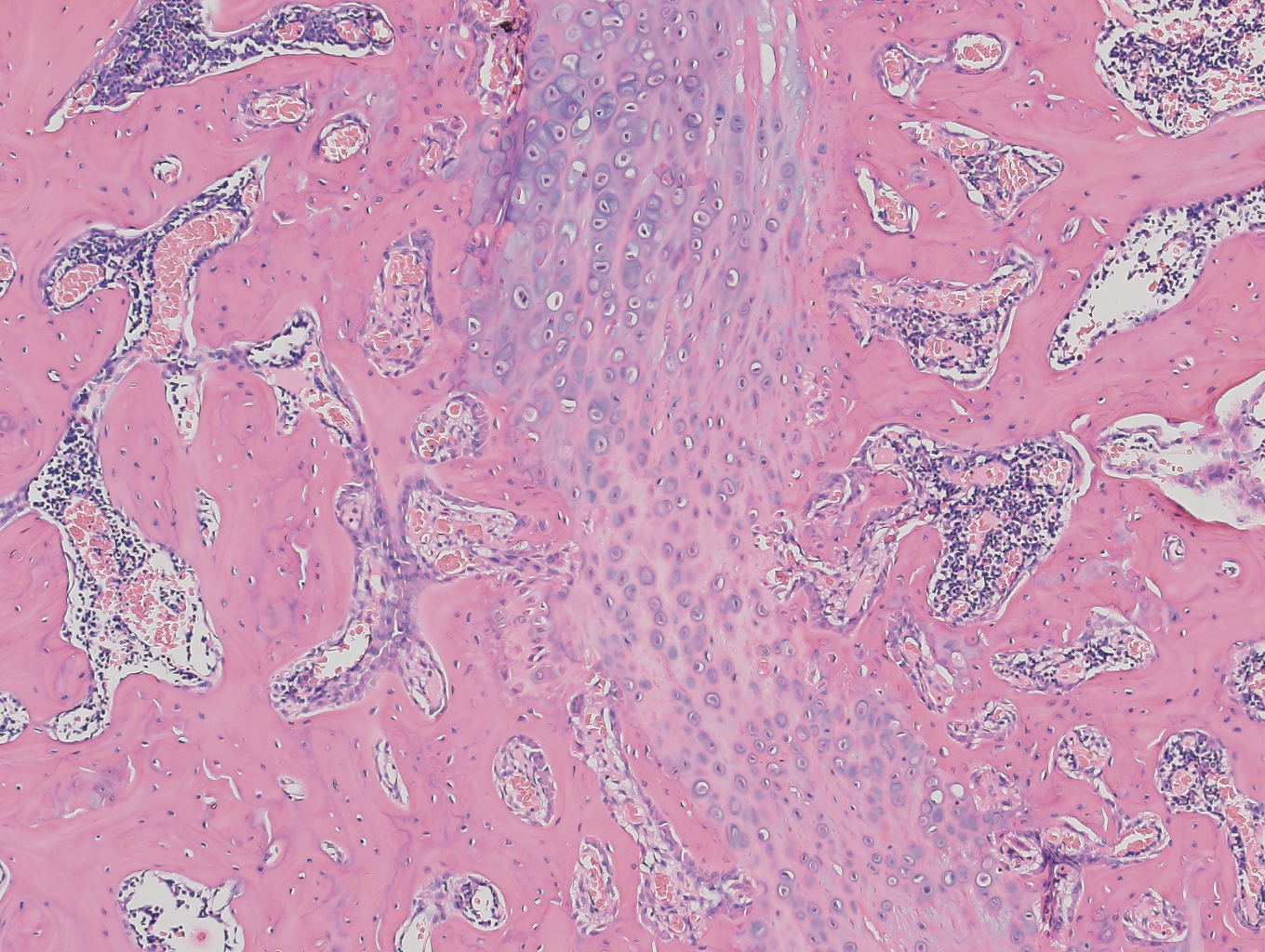

Supplement: Supplementary file 2 [file DataSheet4.ZIP › Figure 6-2 HE/TRX100uM- 6w -2.tif]

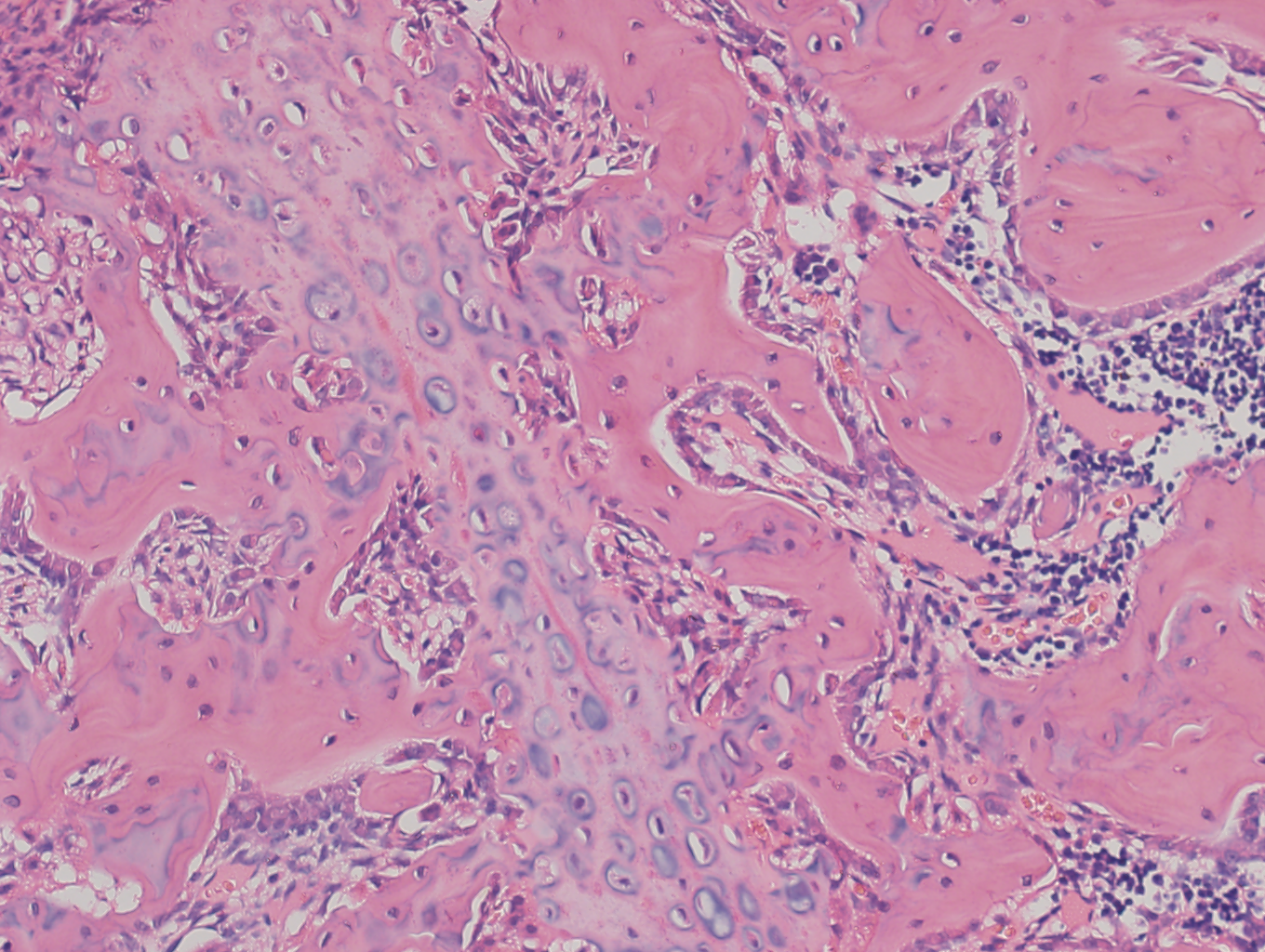

Supplement: Supplementary file 2 [file DataSheet4.ZIP › Figure 6-2 HE/TRX100uM-4W-2.tif]

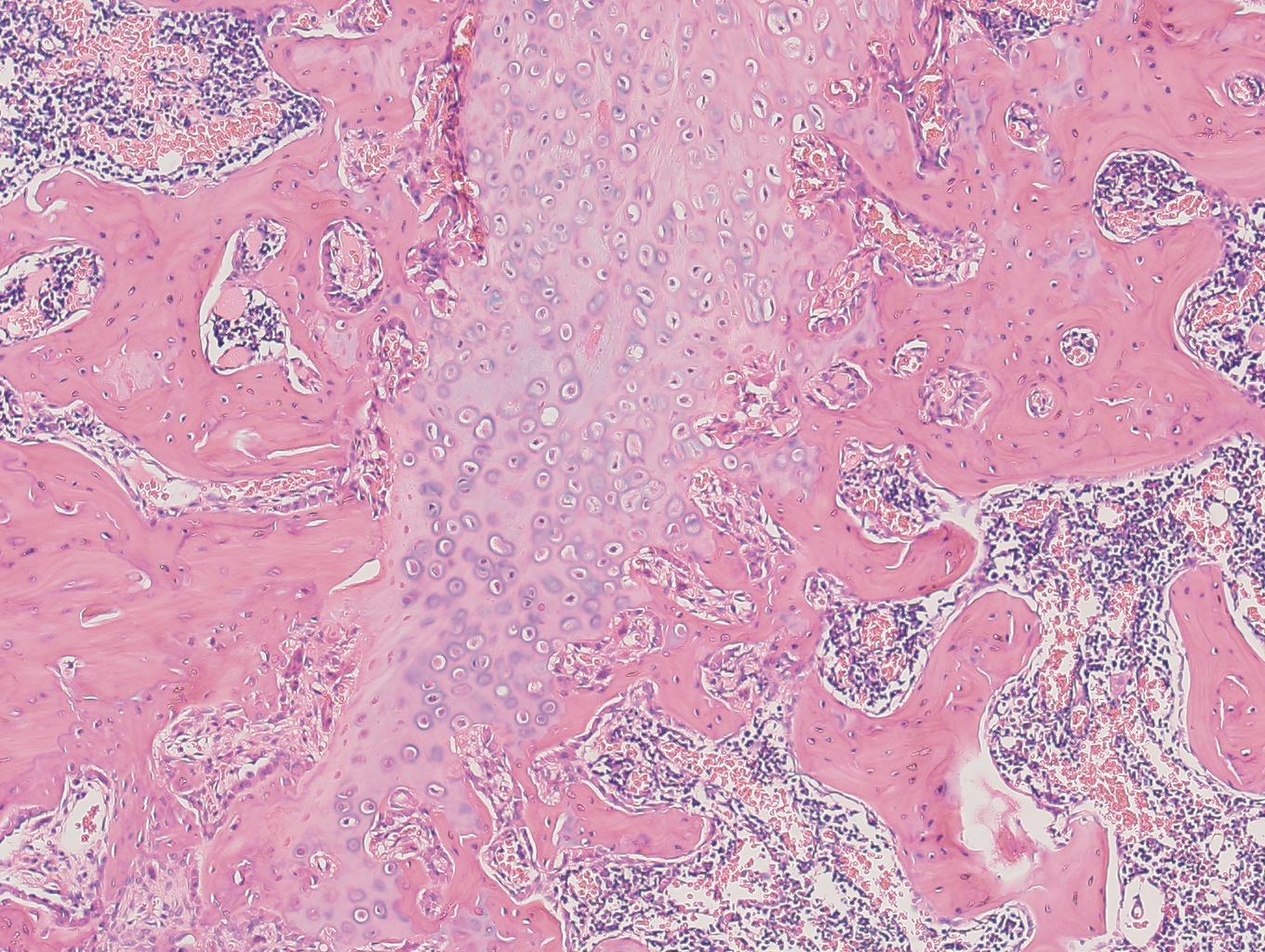

Supplement: Supplementary file 2 [file DataSheet4.ZIP › Figure 6-2 HE/TRX200uM-6W-2.tif]

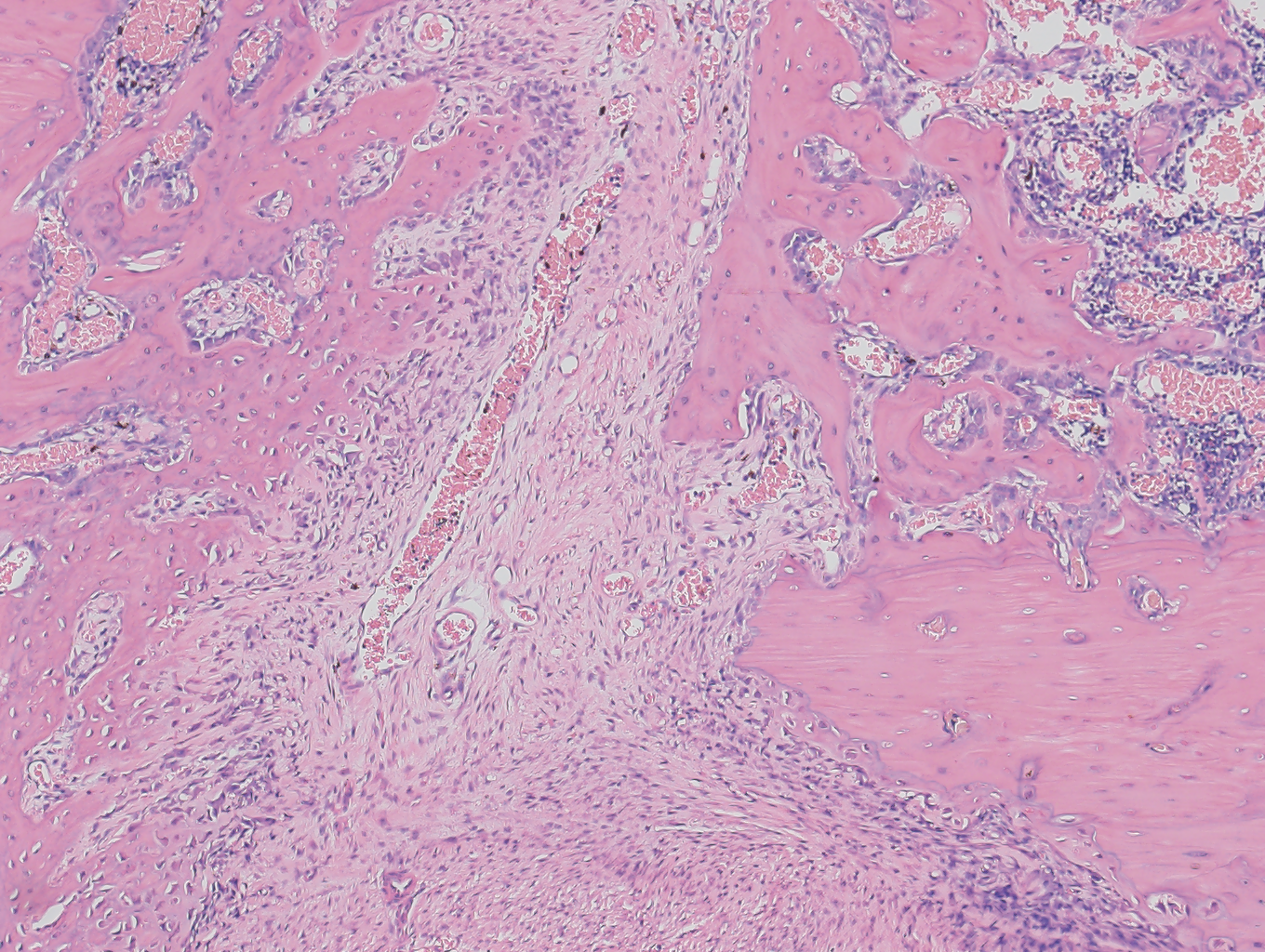

Supplement: Supplementary file 2 [file DataSheet4.ZIP › Figure 6-2 HE/blank 4w -2.tif]

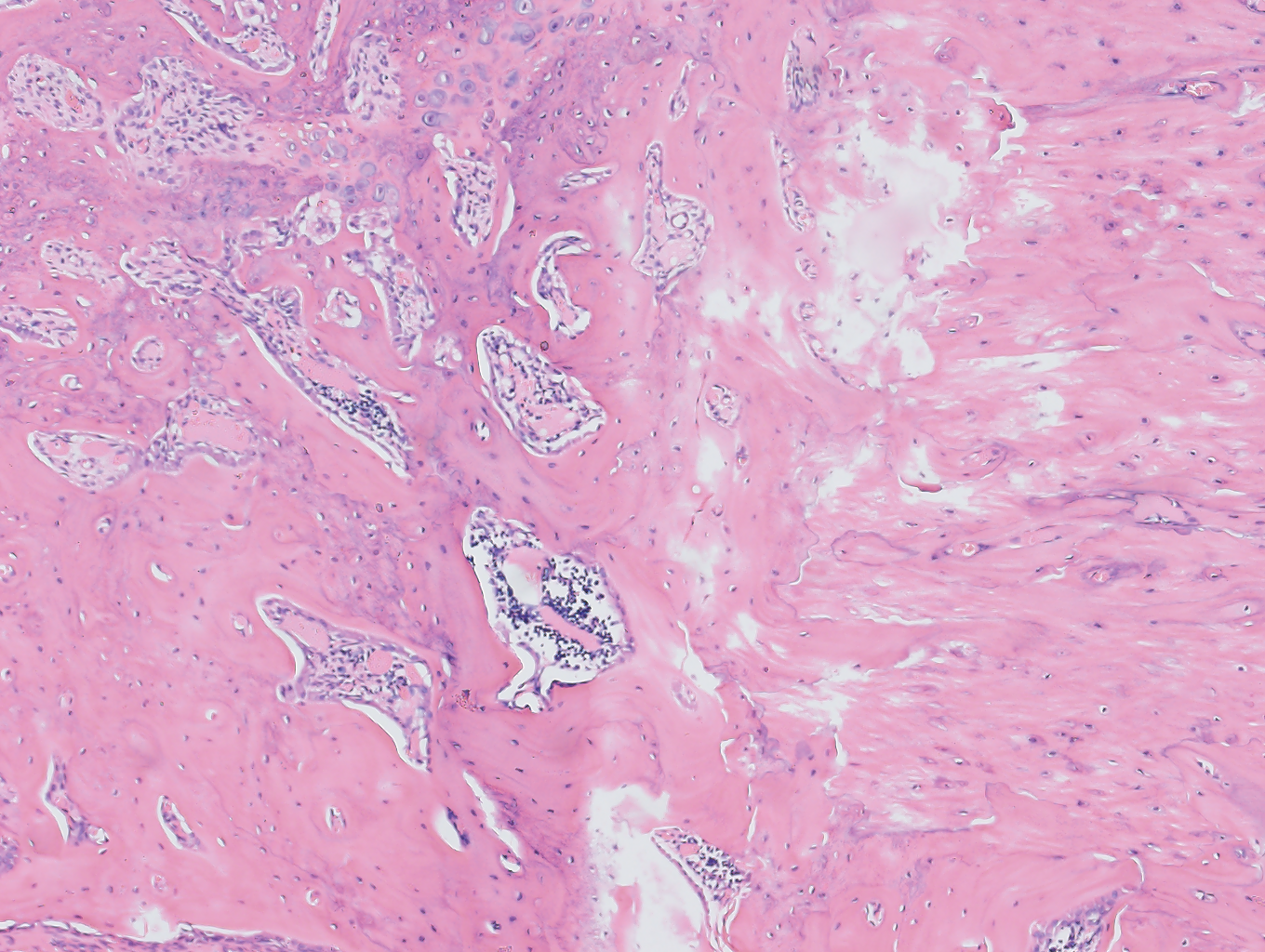

Supplement: Supplementary file 2 [file DataSheet4.ZIP › Figure 6-2 HE/blank-6w-2.tif]

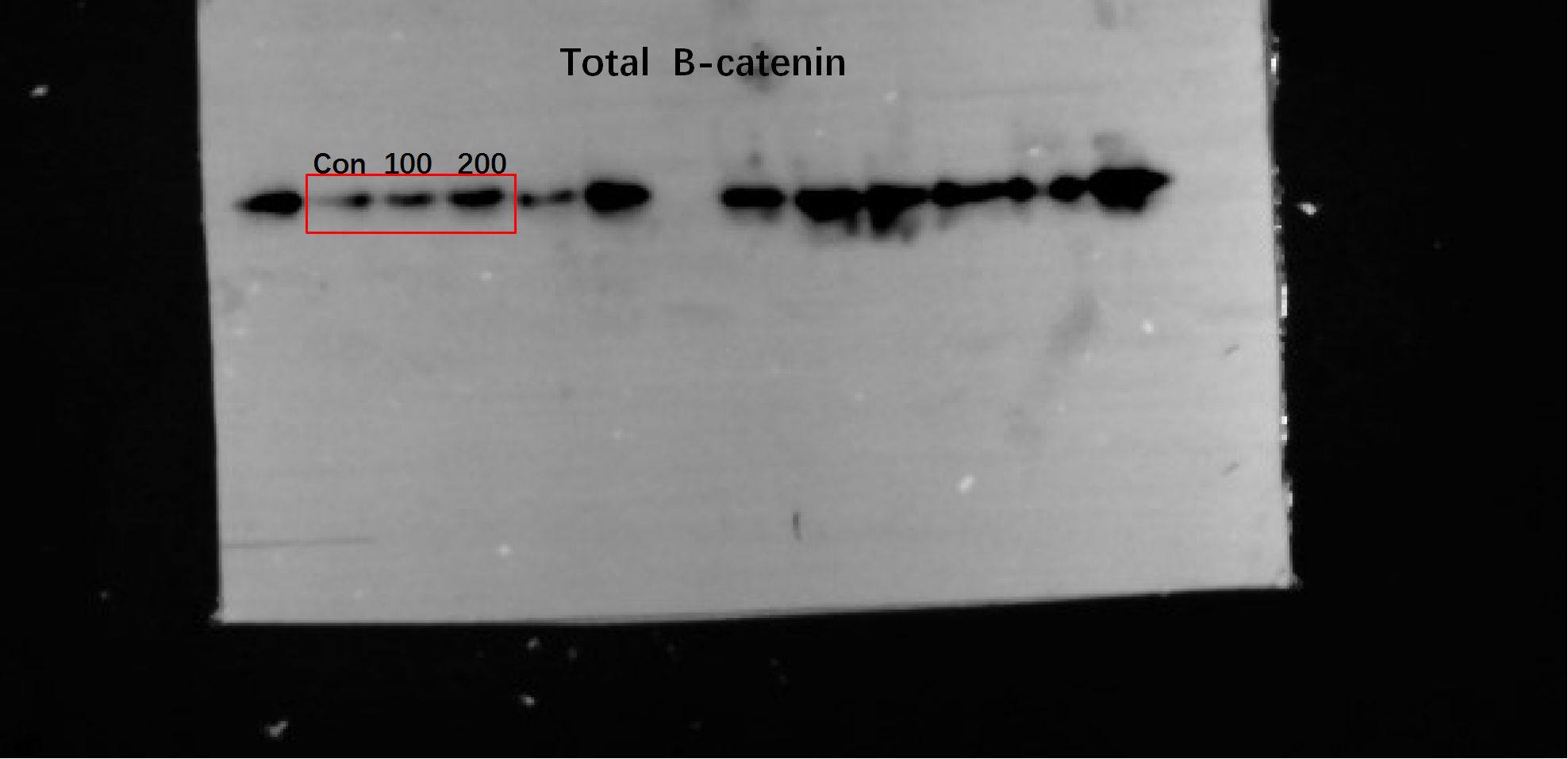

Supplement: Supplementary file 3 [file DataSheet1.ZIP › Figure3B WB/Figure 3B-Total B-catenin.tif]

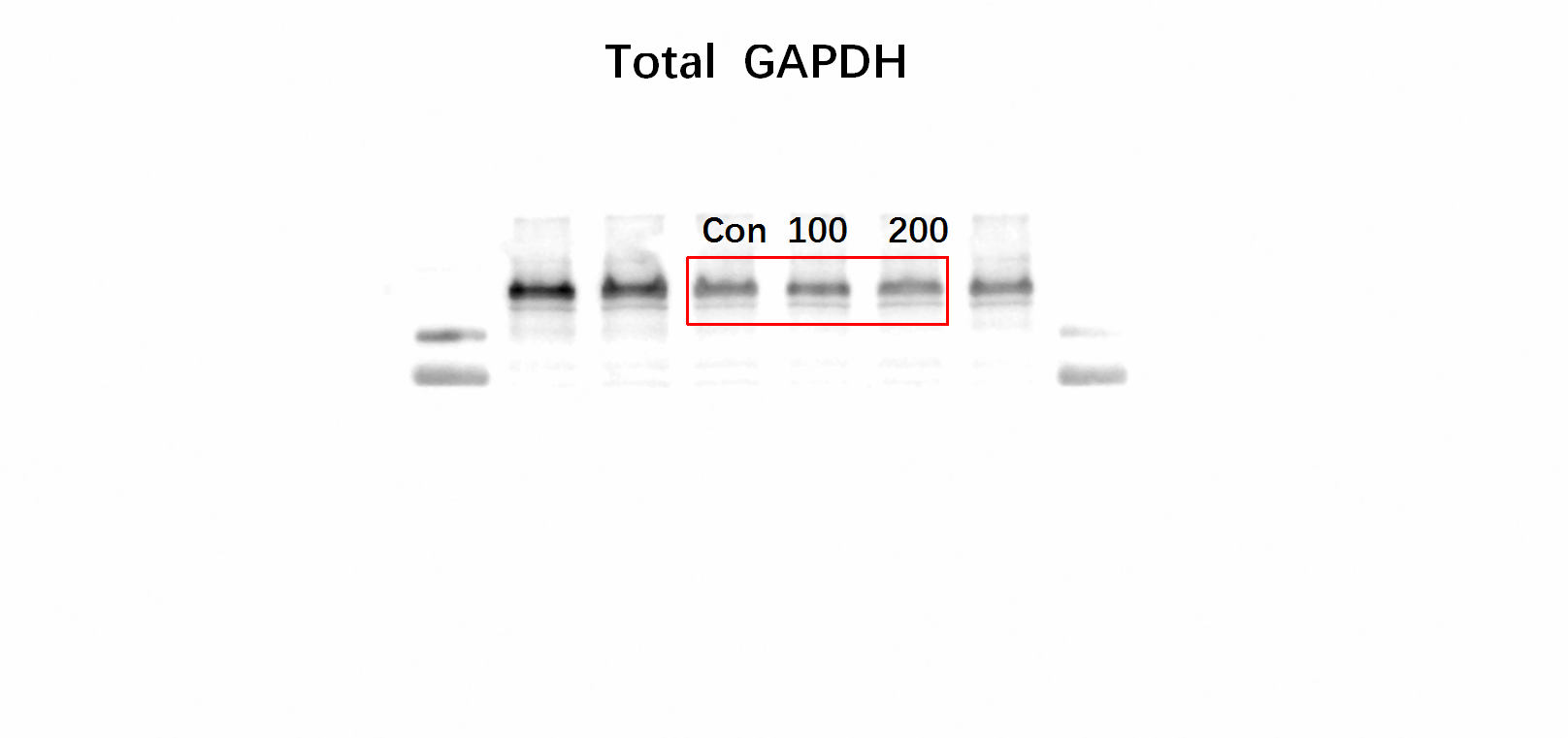

Supplement: Supplementary file 3 [file DataSheet1.ZIP › Figure3B WB/Figure 3B-Total GAPDH.tif]

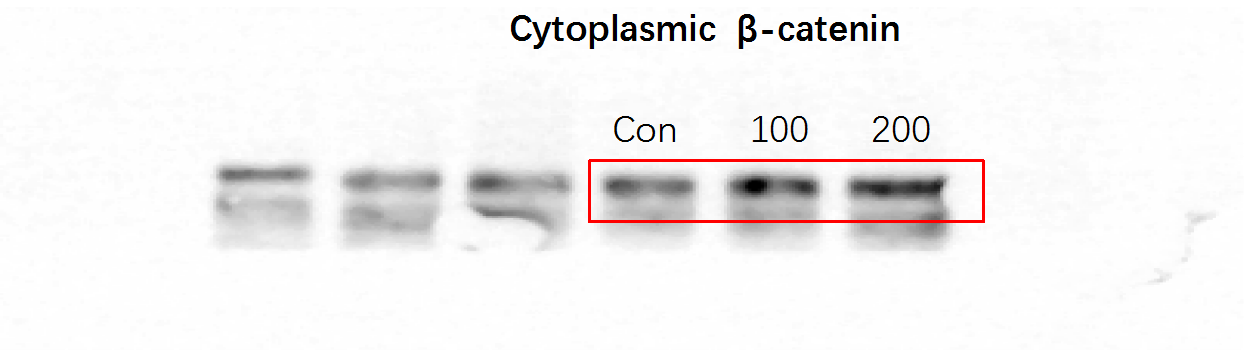

Supplement: Supplementary file 3 [file DataSheet1.ZIP › Figure3B WB/Figure 3B-cytoplasm B-catenin.tif]

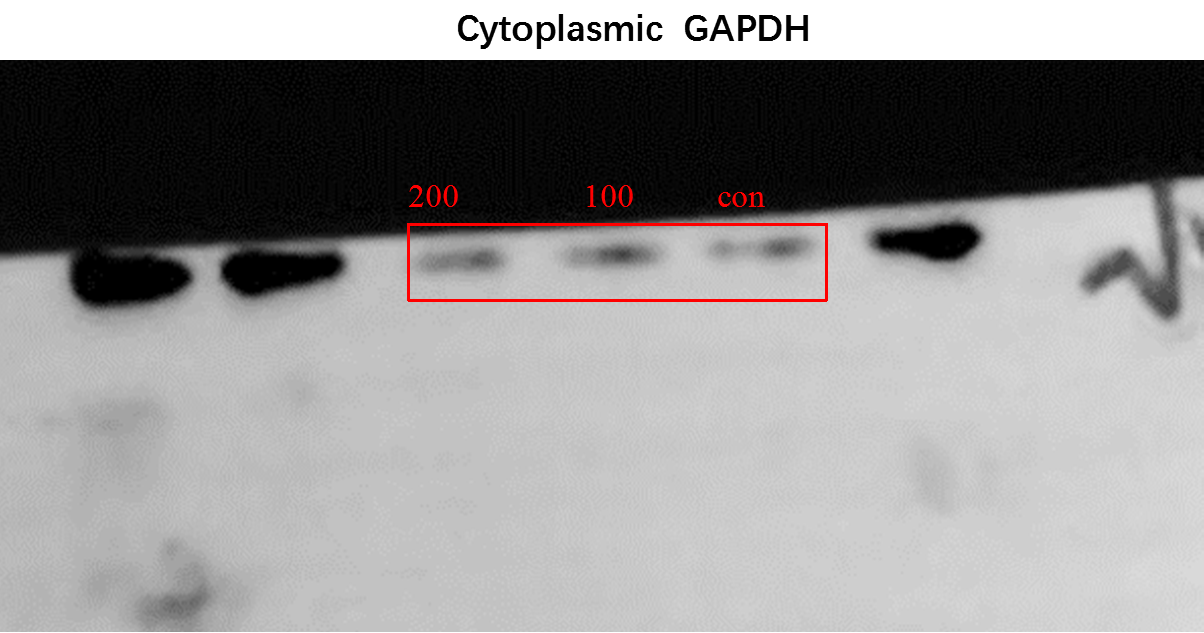

Supplement: Supplementary file 3 [file DataSheet1.ZIP › Figure3B WB/Figure 3B-cytoplasm GAPDH.tif]

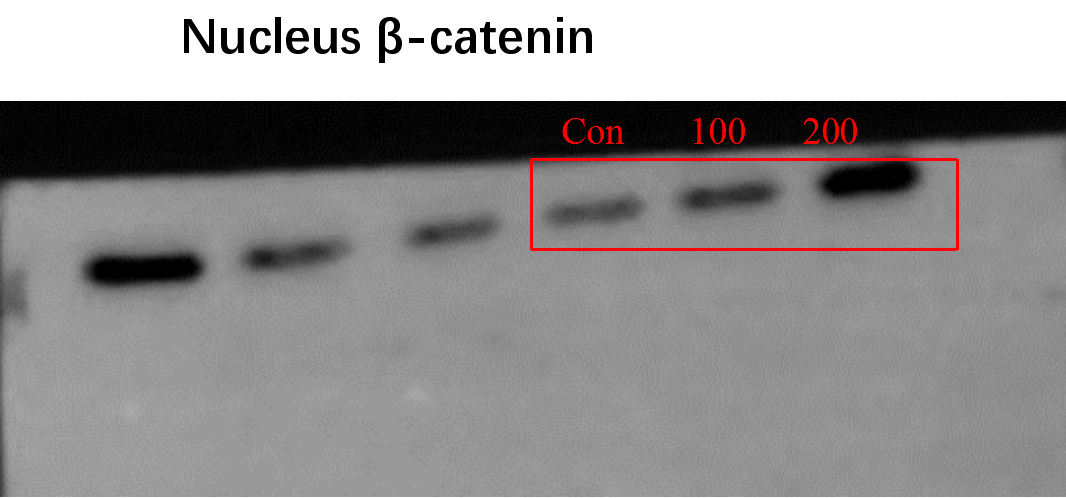

Supplement: Supplementary file 3 [file DataSheet1.ZIP › Figure3B WB/Figure 3B-nucleic B-catenin.tif]

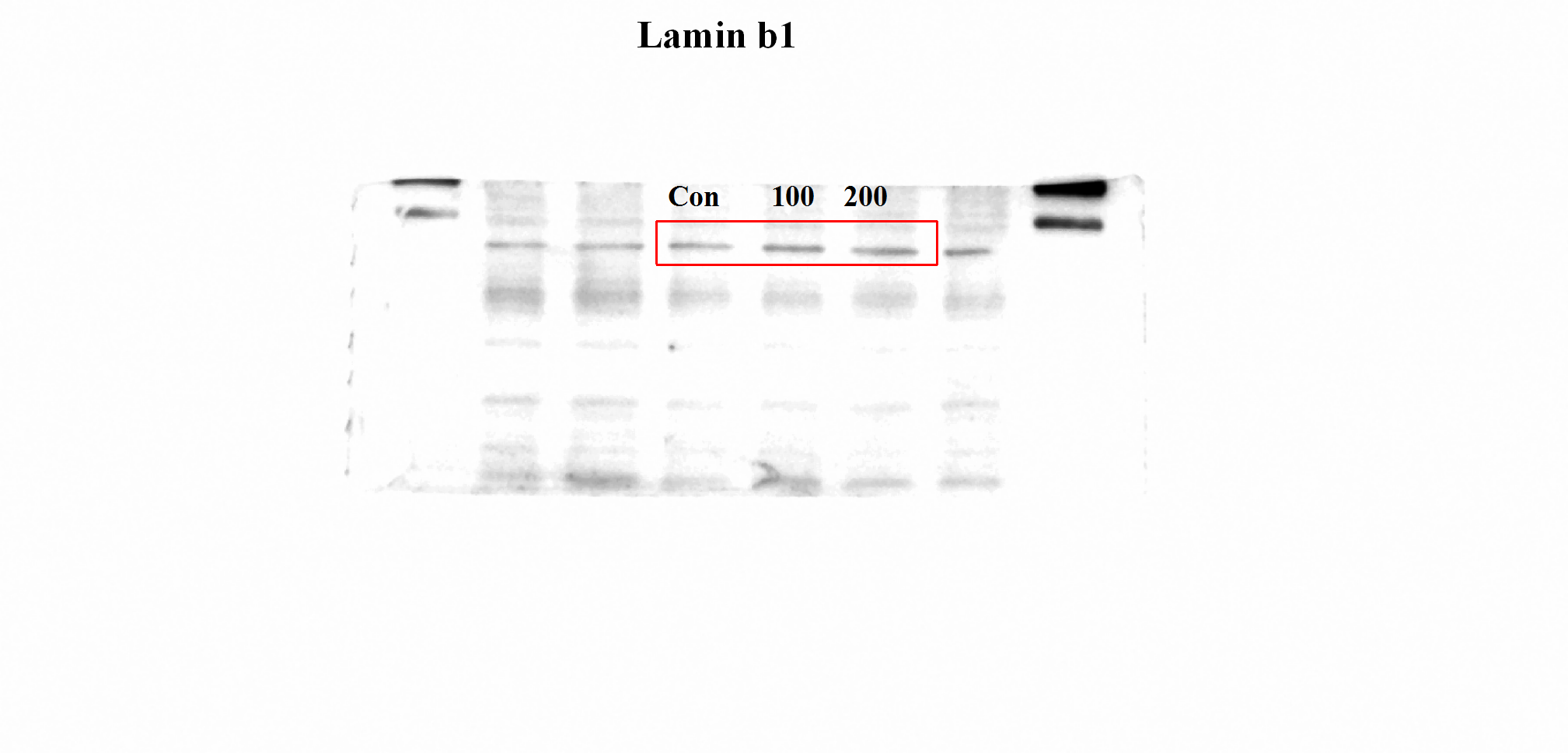

Supplement: Supplementary file 3 [file DataSheet1.ZIP › Figure3B WB/Figure 3B-nucleic Laminb1.tif]

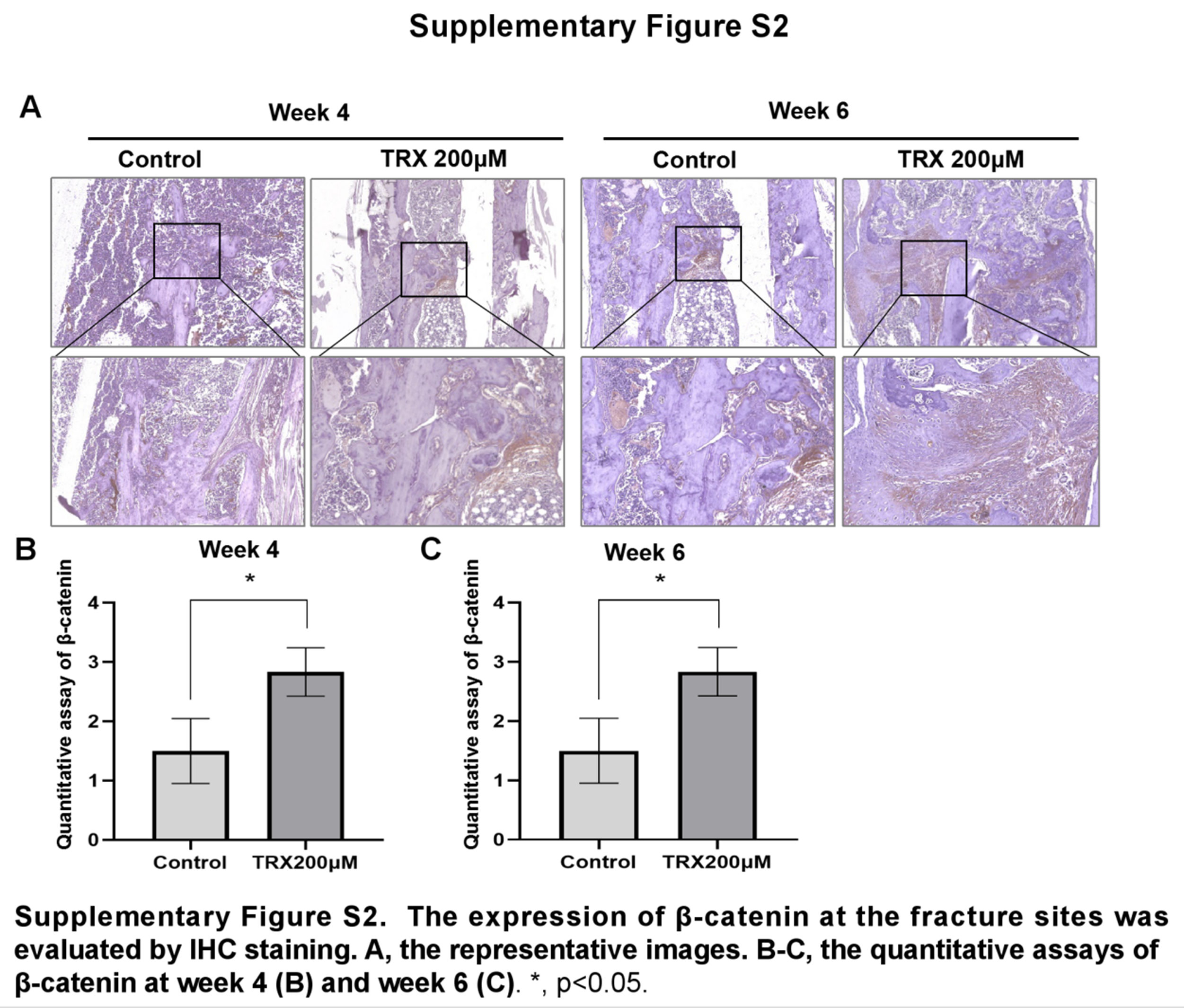

Supplement: Supplementary file 4 [file Image2.TIF]

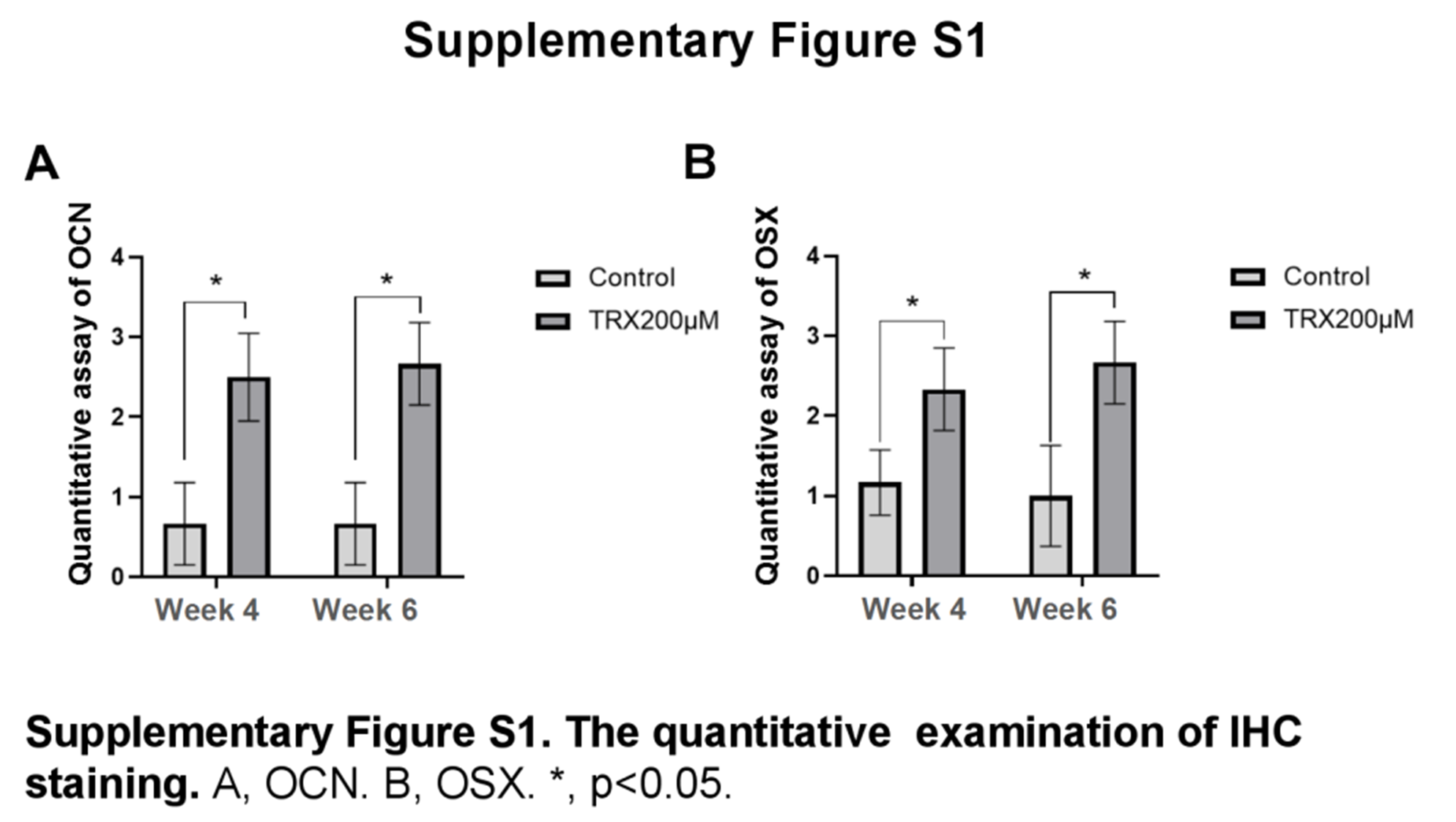

Supplement: Supplementary file 5 [file Image1.TIF]

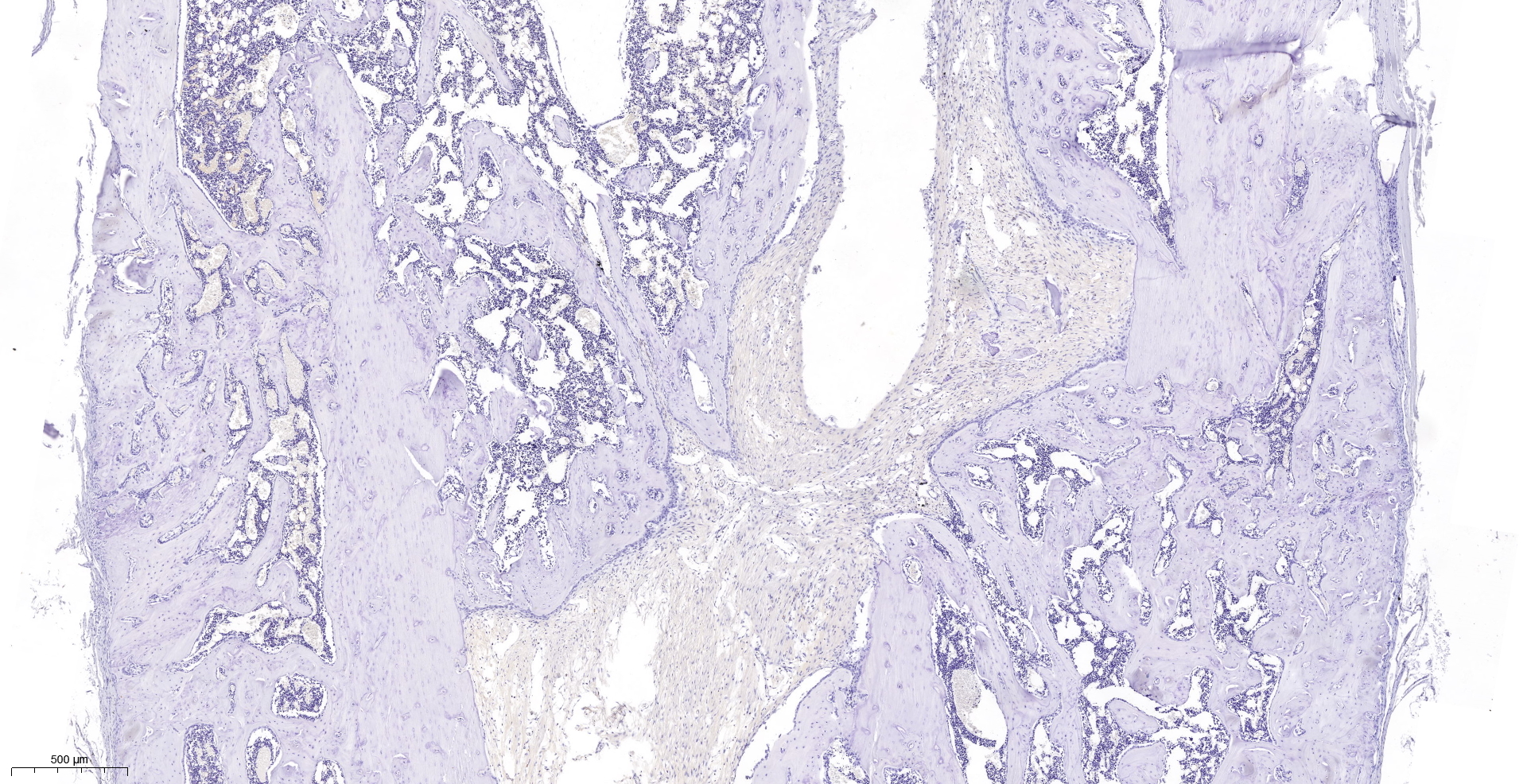

Supplement: Supplementary file 6 [file DataSheet2.ZIP › Figure 7 IHC/OCN-4W-Control-1.jpg]

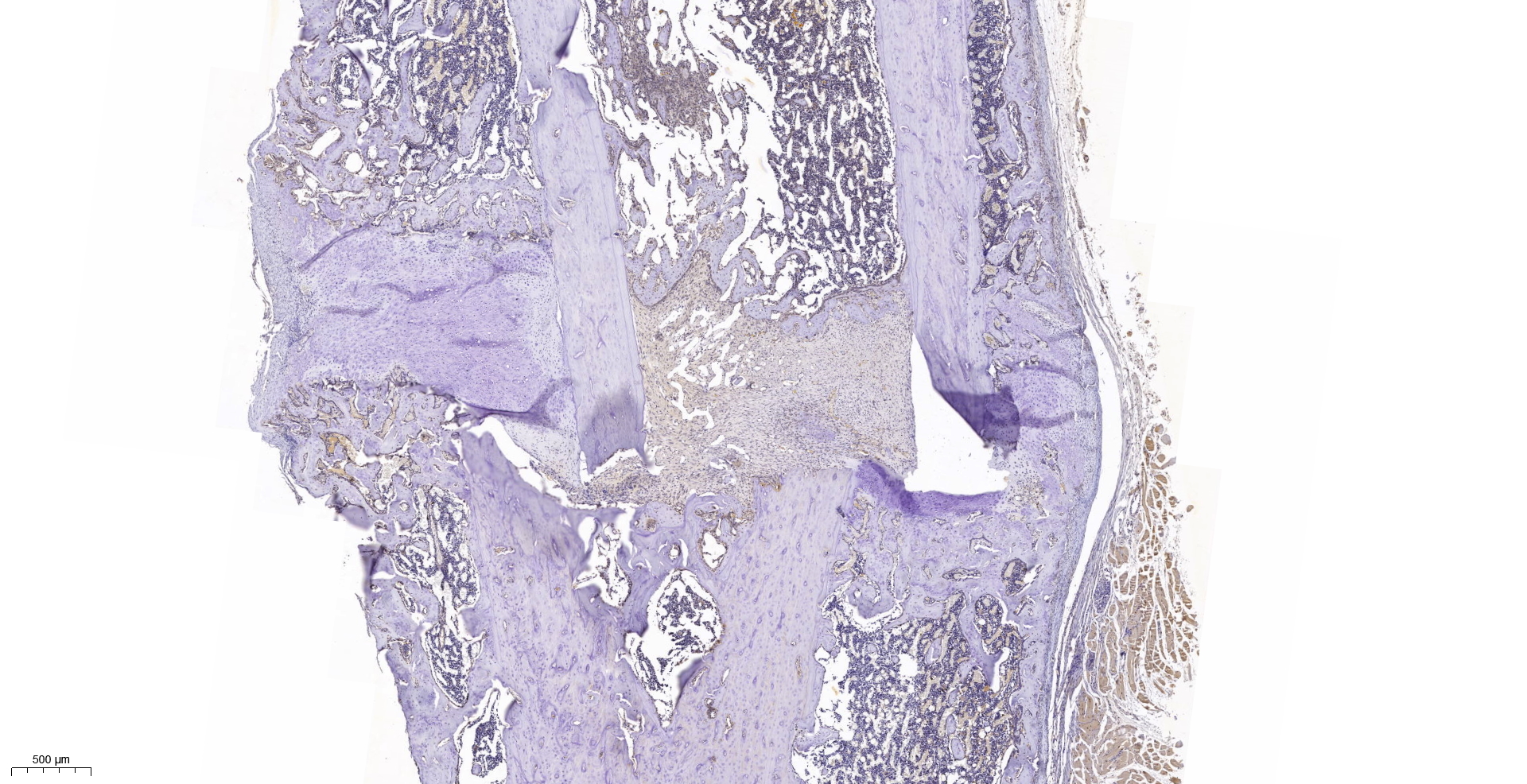

Supplement: Supplementary file 6 [file DataSheet2.ZIP › Figure 7 IHC/OCN-4W-TRX200uM-1.jpg]

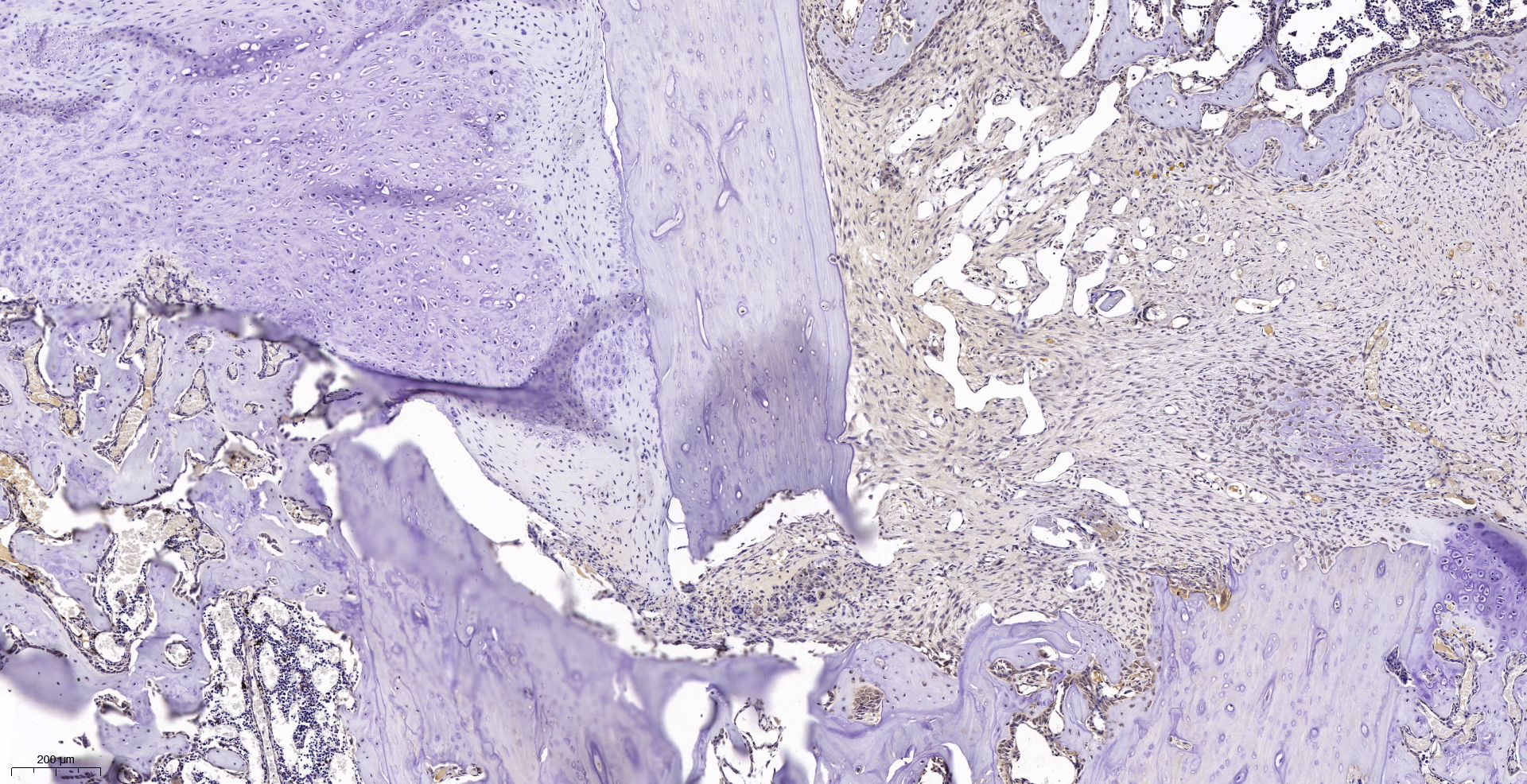

Supplement: Supplementary file 6 [file DataSheet2.ZIP › Figure 7 IHC/OCN-4W-TRX200uM-2.jpg]

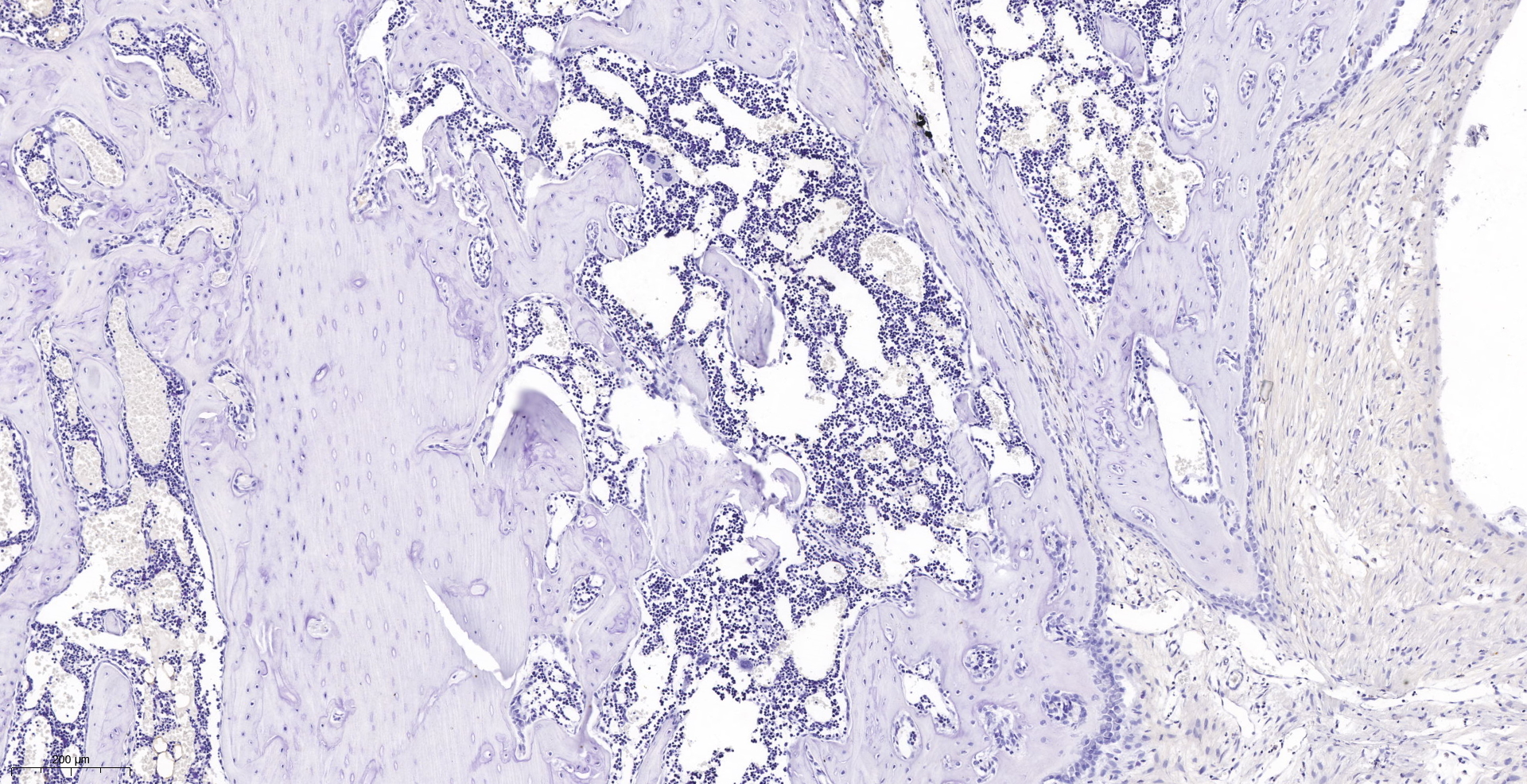

Supplement: Supplementary file 6 [file DataSheet2.ZIP › Figure 7 IHC/OCN-4W-control-2.jpg]

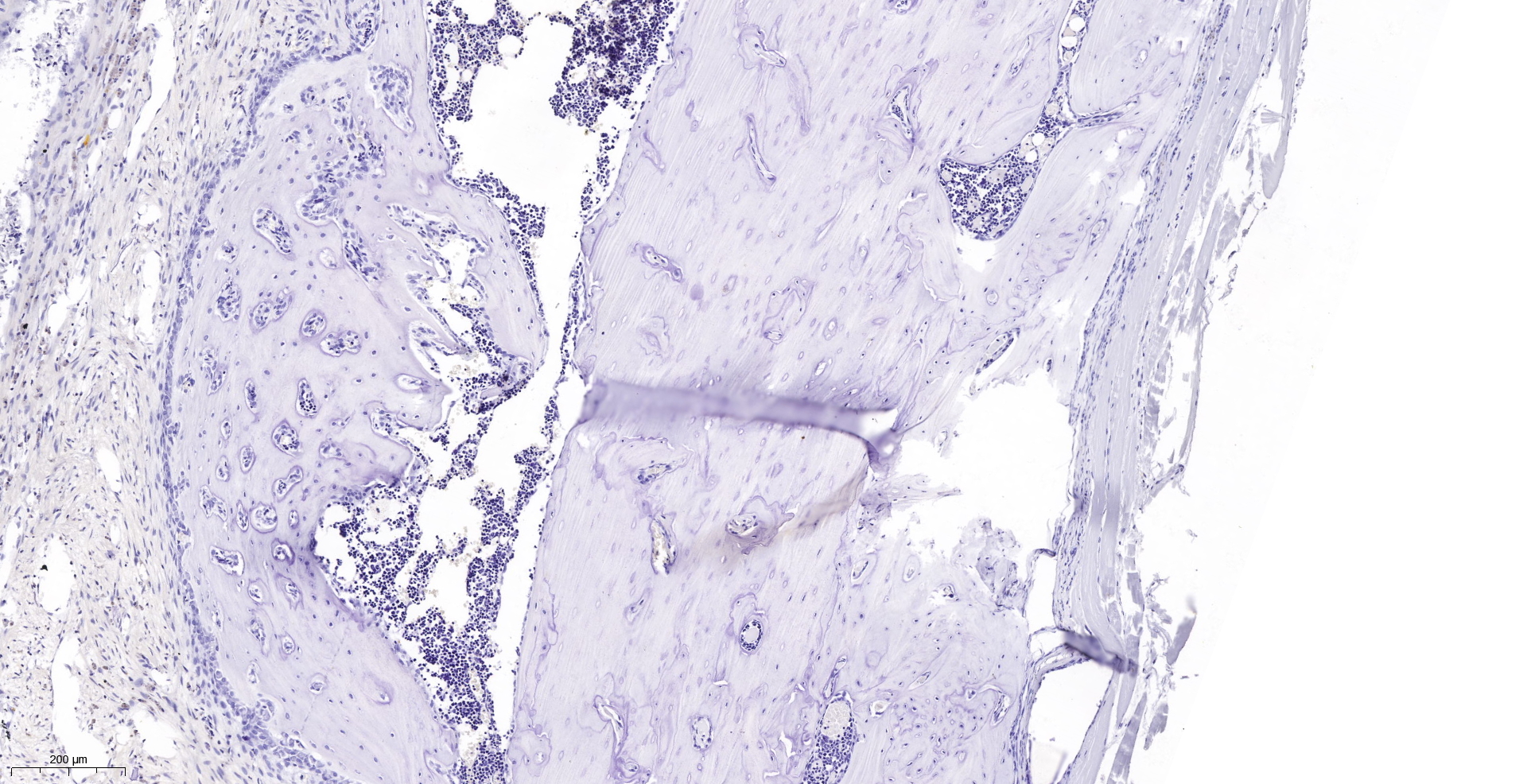

Supplement: Supplementary file 6 [file DataSheet2.ZIP › Figure 7 IHC/OCN-6W-Control-2.jpg]

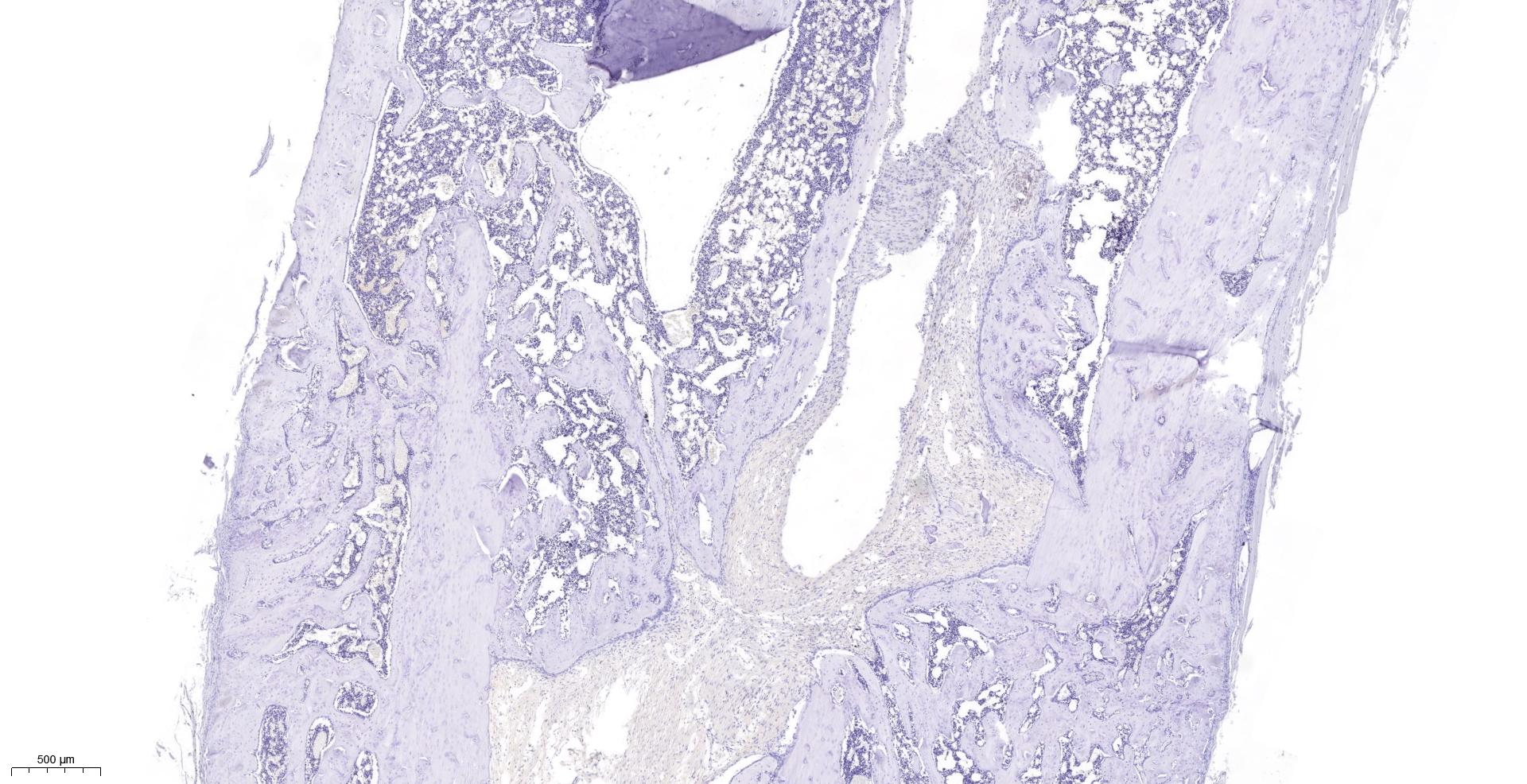

Supplement: Supplementary file 6 [file DataSheet2.ZIP › Figure 7 IHC/OCN-6W-Control1.jpg]

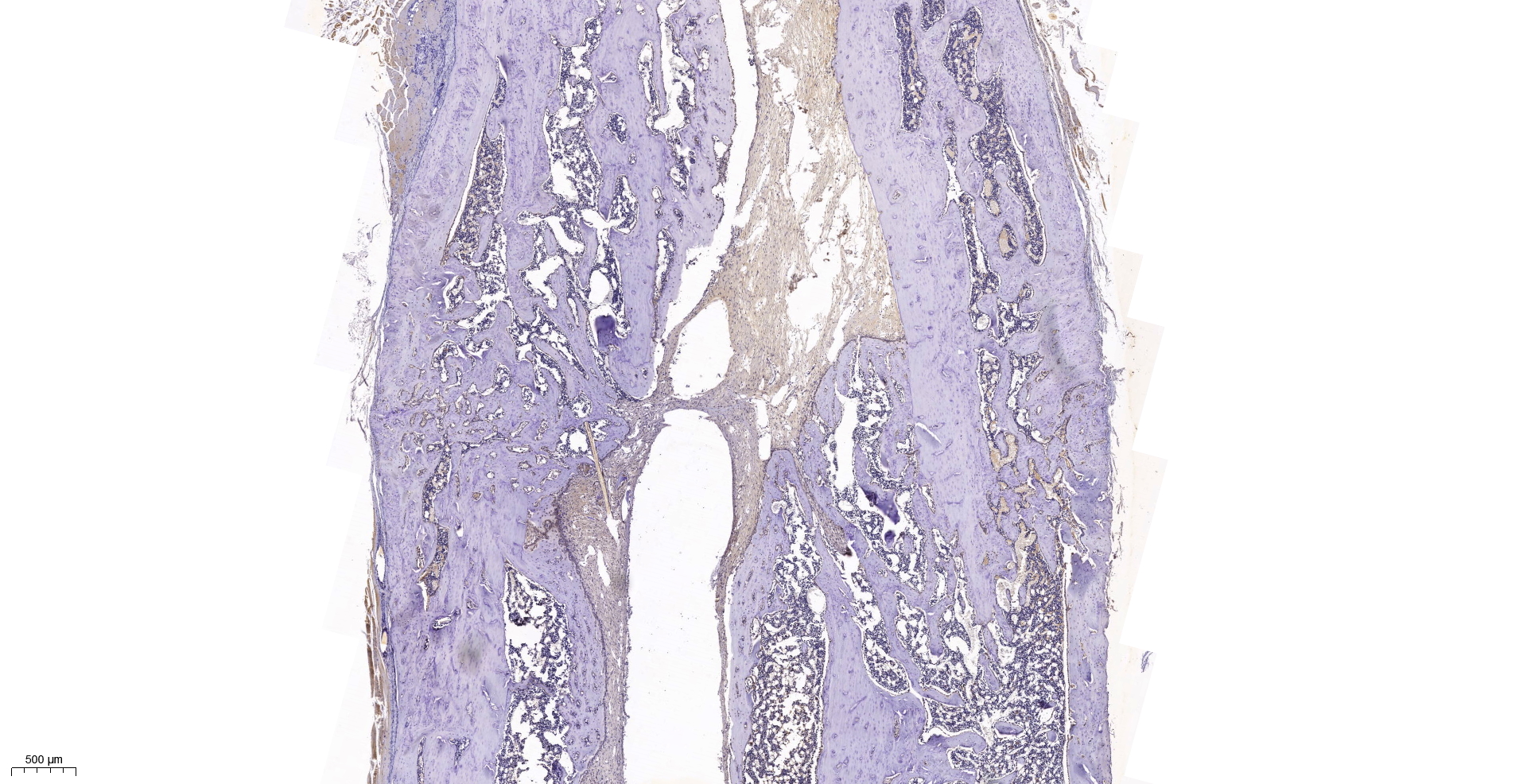

Supplement: Supplementary file 6 [file DataSheet2.ZIP › Figure 7 IHC/OCN-6W-TRX200uM-1.jpg]

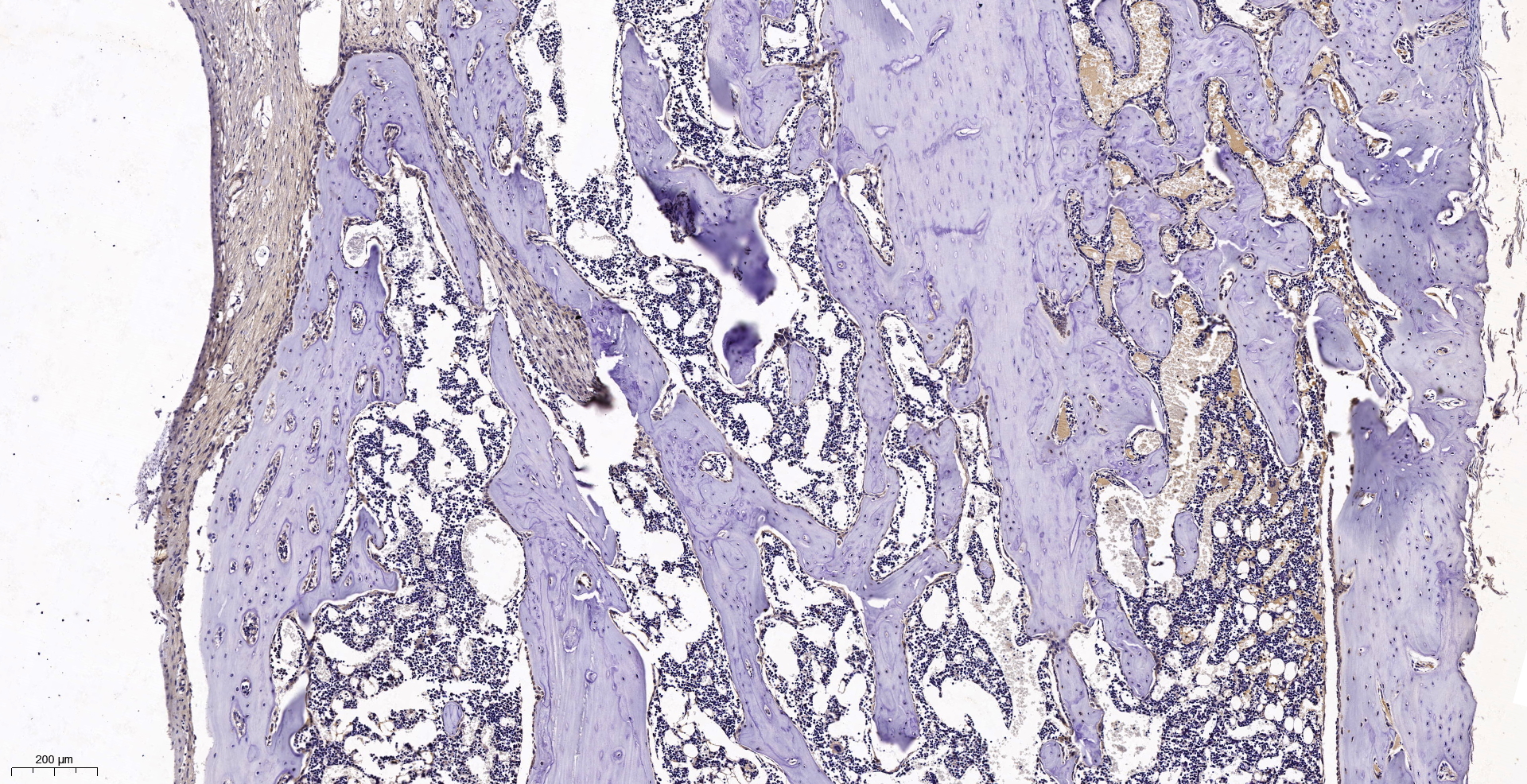

Supplement: Supplementary file 6 [file DataSheet2.ZIP › Figure 7 IHC/OCN-6W-TRX200uM-2.jpg]

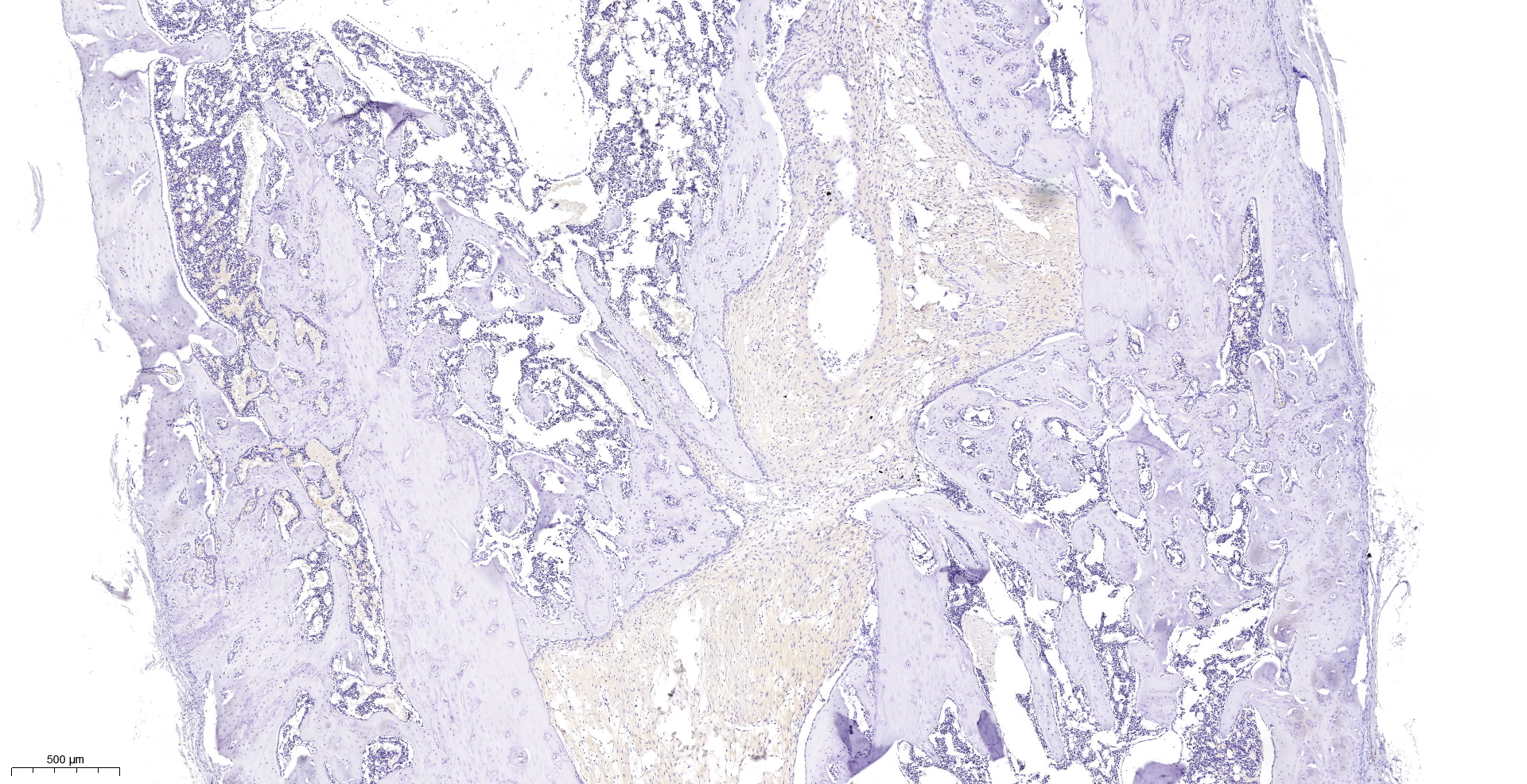

Supplement: Supplementary file 6 [file DataSheet2.ZIP › Figure 7 IHC/OSX-4W-Control1.jpg]

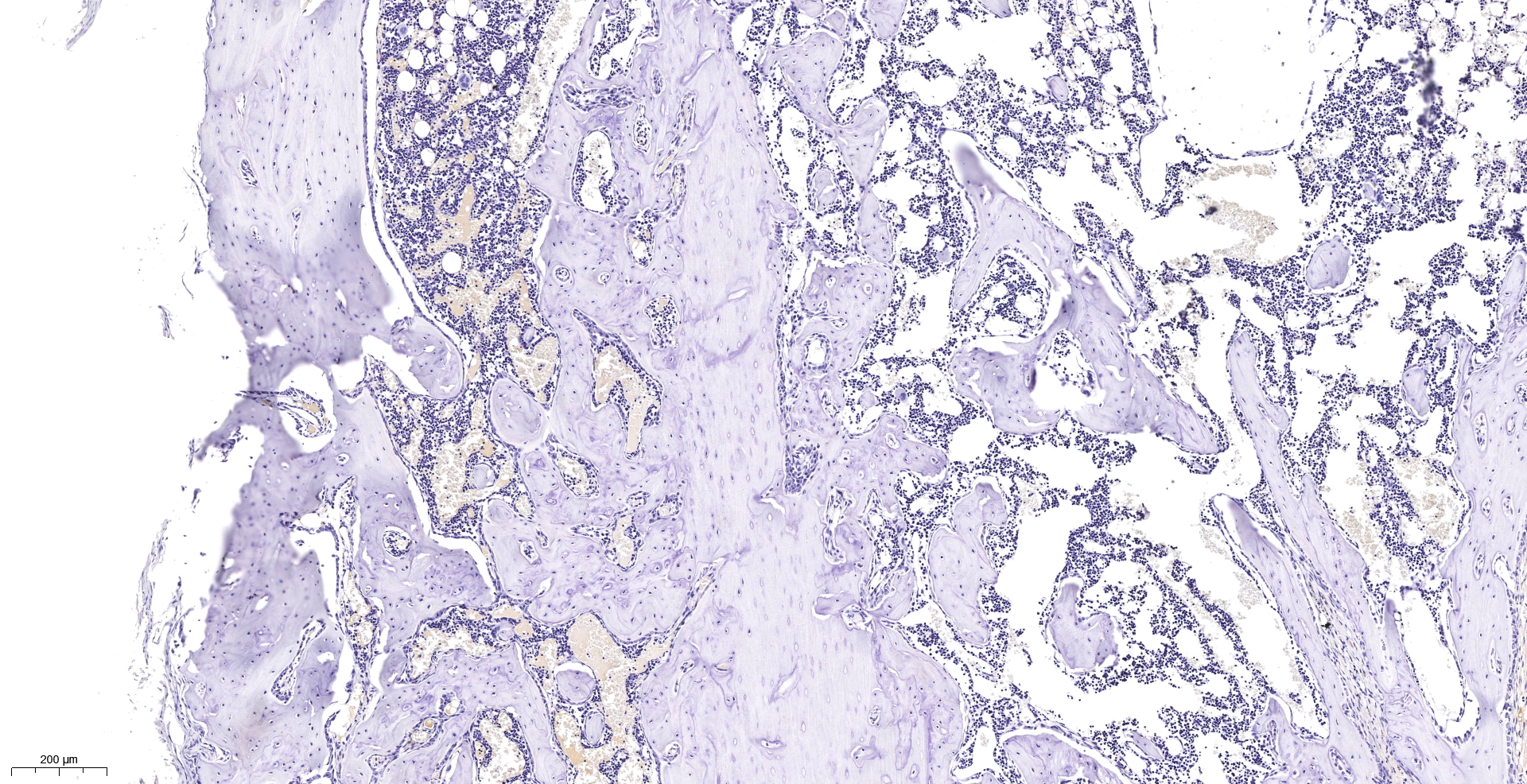

Supplement: Supplementary file 6 [file DataSheet2.ZIP › Figure 7 IHC/OSX-4W-Control2.jpg]

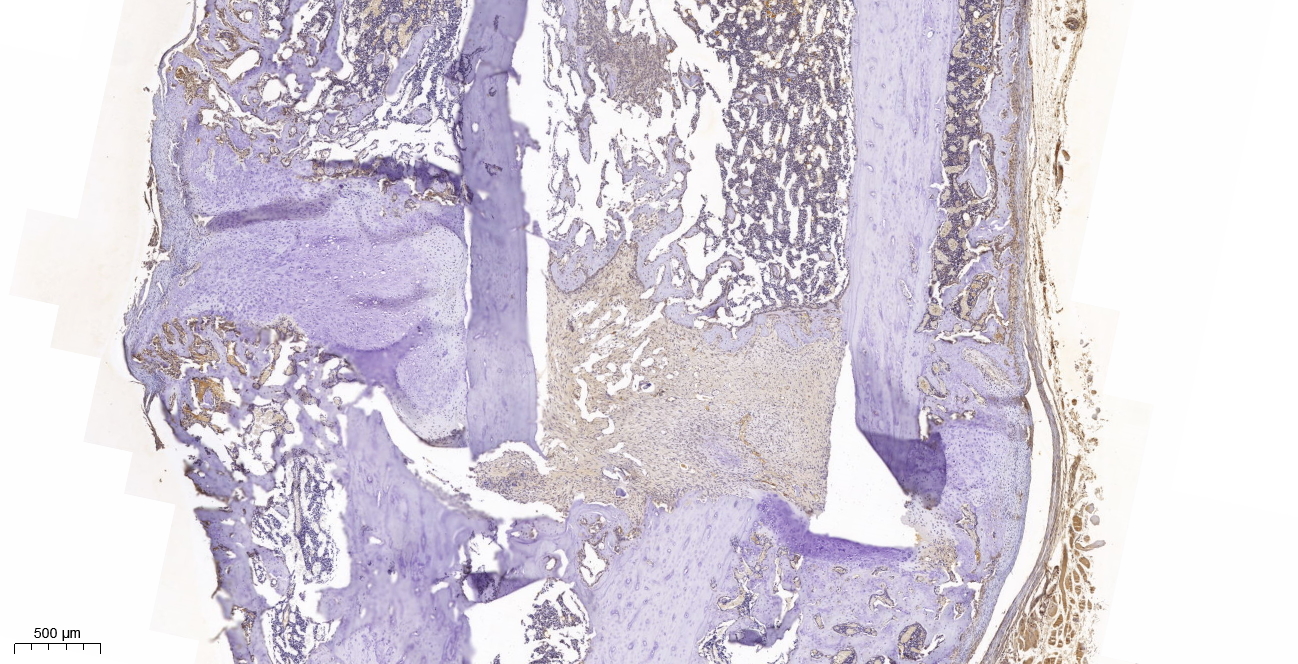

Supplement: Supplementary file 6 [file DataSheet2.ZIP › Figure 7 IHC/OSX-4W-TRX200uM-1.jpg]

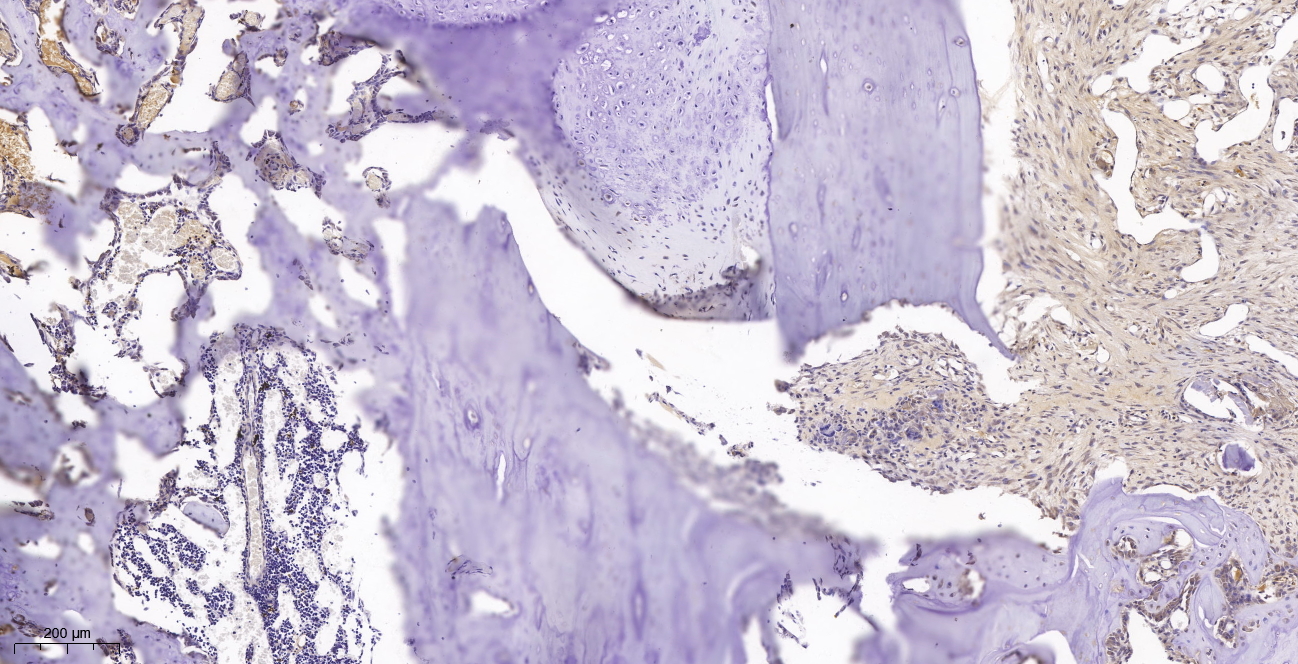

Supplement: Supplementary file 6 [file DataSheet2.ZIP › Figure 7 IHC/OSX-4W-TRX200uM-2.jpg]

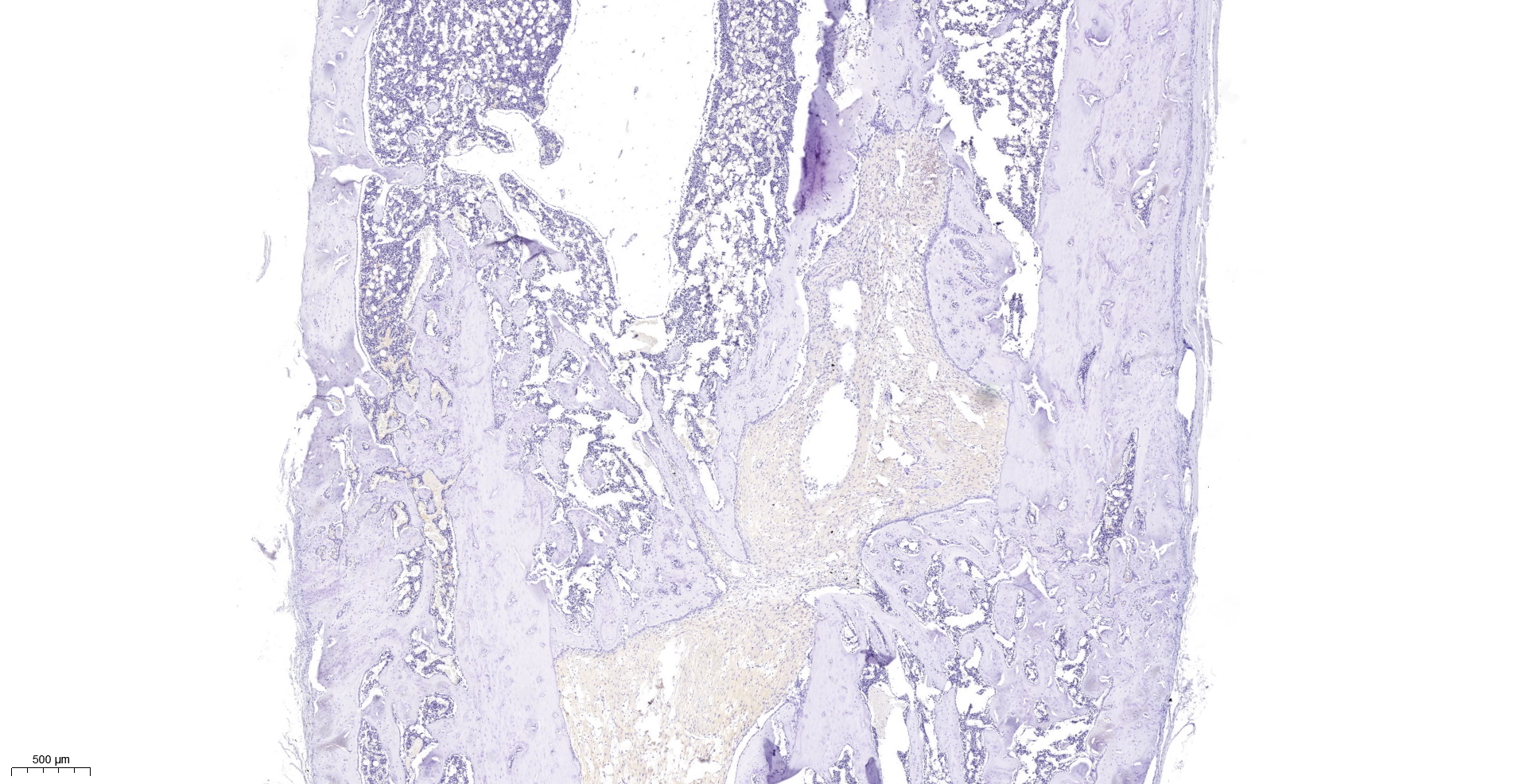

Supplement: Supplementary file 6 [file DataSheet2.ZIP › Figure 7 IHC/OSX-6W-Control.jpg]

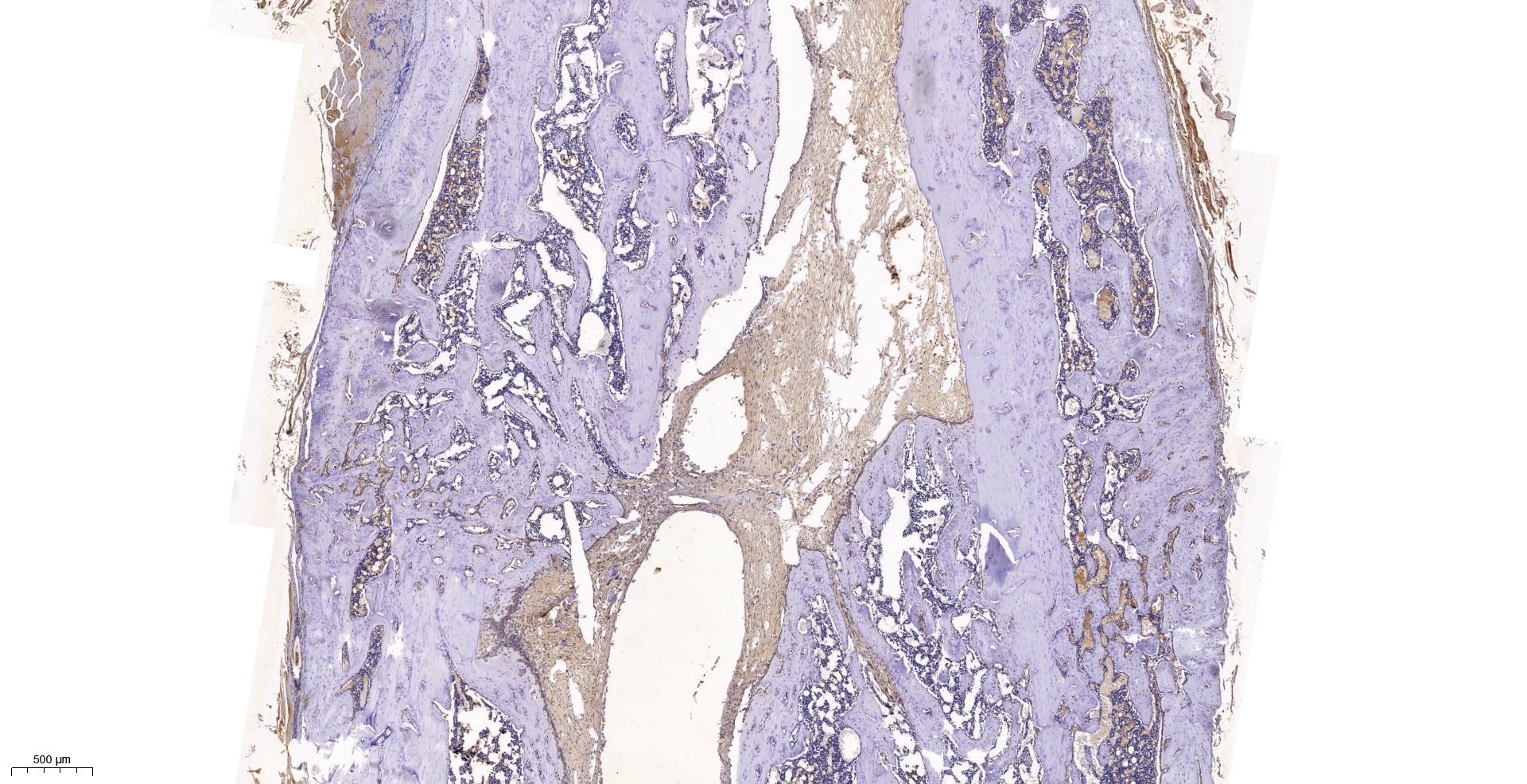

Supplement: Supplementary file 6 [file DataSheet2.ZIP › Figure 7 IHC/OSX-6W-TRX200uM-1.jpg]

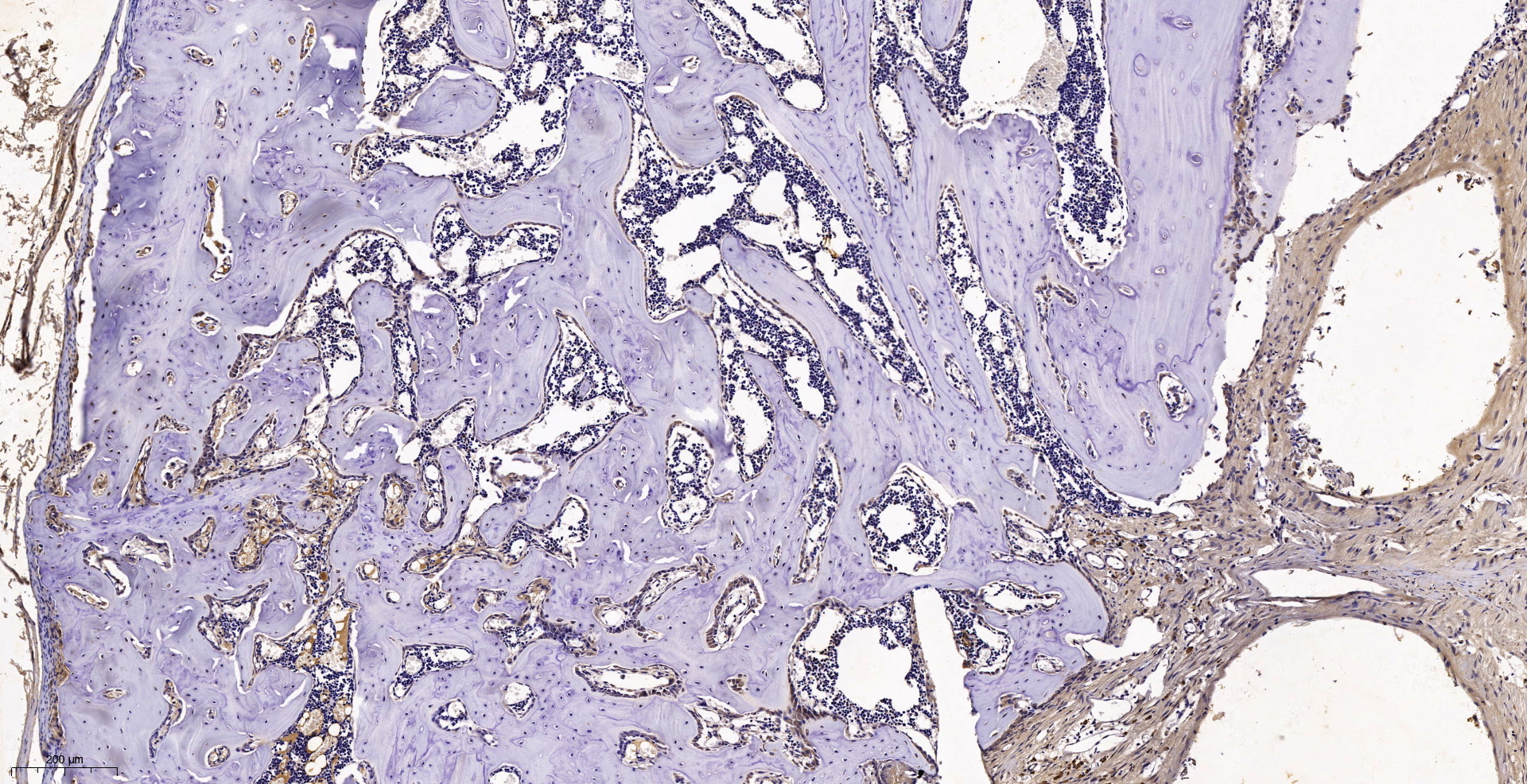

Supplement: Supplementary file 6 [file DataSheet2.ZIP › Figure 7 IHC/OSX-6W-TRX200uM-2.jpg]

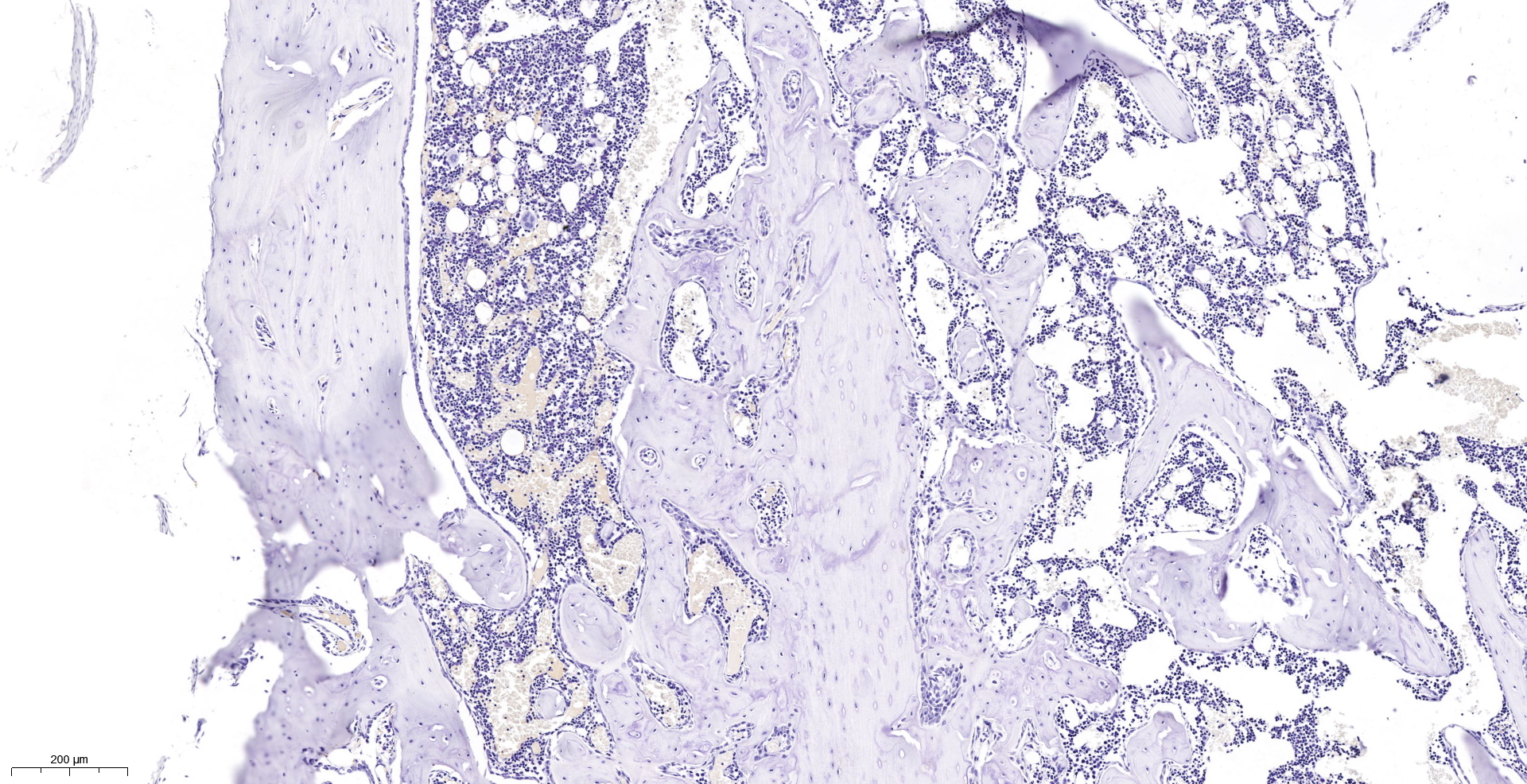

Supplement: Supplementary file 6 [file DataSheet2.ZIP › Figure 7 IHC/OSX-6W-control2.jpg]
